# Precipitation modeling in Northeastern Bangladesh–India transboundary flood regions using bi-metaheuristic-optimized NMF-neural network

**DOI:** 10.1038/s41598-025-22135-7

**Published:** 2025-10-31

**Authors:** Shuvendu Pal Shuvo, Shirshendu Pal Shibazee, Chaitee Das, Ankita Bhowmick Oishi

**Affiliations:** 1 Department of Civil Engineering , Khulna University of Engineering and Technology (KUET) , Khulna, Bangladesh; 2 Department of Electrical and Electronic Engineering , Bangladesh University of Engineering and Technology , Dhaka, Bangladesh; 3 Department of Materials Science and Engineering , Khulna University of Engineering & Technology , Khulna, Bangladesh; 4 Department of Leather Engineering , Khulna University of Engineering & Technology , Khulna, Bangladesh

**Keywords:** Rainfall, Metaheuristics optimization, Hybridization, Non-negative matrix factorization (NMF), Artificial neural network, Climate sciences, Hydrology

## Abstract

Two fundamental questions in rainfall modeling using Artificial Neural Networks (ANN) are: (1) how to best handle highly nonlinear, noisy, short-duration rainfall data, and (2) how to locate a solution to the optimization issue of ANN, particularly in formulating the optimum weights and biases. Most of the single-step rainfall forecasting models that rely on single-step optimization and conventional ANN feature selection techniques are not capable of adequately addressing these critical challenges. In this study, Non-Negative Matrix Factorization (NMF) is applied to model complex short-term rainfall patterns, and an optimal differential approach is used to compute NMF components. For the optimization of the ANN model, in this paper, three recently proposed metaheuristic algorithms namely Harris Hawks Optimization (HHO), Egret Swarm Optimization Algorithm (ESOA), and Hippopotamus Optimization (HO) are explored along with traditional well-known methods like Genetic Algorithm (GA) and Particle Swarm Optimization (PSO). Furthermore, a two-step hybrid optimization approach is introduced to enhance ANN performance in rainfall forecasting. This significantly improves model precision, with reductions in mean squared error (MSE) ranging from 1.00% to 97.46% in Sylhet, 8.33% to 97.10% in Chittagong, 6.96% to 89.57% in Meghalaya, and 17.74% to 76.09% in Assam compared to single-step optimization models. The results indicate that the two-step optimization approach not only outperforms single optimization techniques but also outperforms recently introduced metaheuristic algorithms such as HHO, ESOA, and HO, demonstrating its superior potential for improving the accuracy of rainfall modeling. On the other hand, the sensitivity analysis of the hidden neuron indicates that the impact of hidden neuron quantity is highly sensitive to the optimizer and the characteristics of the data.

**Supplementary Information:**

The online version contains supplementary material available at 10.1038/s41598-025-22135-7.

## Introduction

Bangladesh, in the Ganges-Brahmaputra Delta, experiences perpetual flooding due to its low elevation much of the nation lies below five meters of sea level and its position at the confluence of three enormous river systems^1^. Normally, 21% of the country, about 31,000 square kilometers, is submerged each year, and intense events overwhelm this by several orders of magnitude, as occurred in 1998 when over two-thirds of the nation was covered by water^2^. This vulnerability is driven by a combination of upstream water inputs responsible for 80% of Bangladesh’s overall streamflow from the Ganges, Brahmaputra Jamuna, and Meghna rivers coupled with flash floods from surrounding hills and intense local precipitation, further boosted by poor drainage, generating a near annual flood cycle^3^. Sylhet, the most water-receiving region of Bangladesh, has an annual rainfall of approximately 4,000 mm, and hence it is highly susceptible to flash and local floods^4^. The district’s topography, surrounded by hills, channels runoff rapidly, overburdening its drainage system. The past of Sylhet’s western region being frequently flooded is seen in records, with the most significant floods taking place in 1781, 1853, 1902, 1966, 1968, 1988, and in recent years, namely 1998, 2000, 2004, 2007, 2010, 2012, 2015, 2016, 2017, 2019, and 2022^4^. Heavy rainfall in Assam and Meghalaya also worsens flooding in Sylhet^5^. Similarly, along the southeast coast, Chittagong, which is one of the key urban centers in the country, suffers from crucial urban flooding primarily due to excess rainfall. Waterlogging during the monsoon is a perpetual problem, covering primary roads, alleys, and business hubs of old and new parts of the city, interrupting day-to-day life and freezing economic activity in the business capital of the nation^6^. With such apocalyptic socio-economic impacts of these floods, accurate prediction of rainfall in Sylhet (Bangladesh), Chittagong (Bangladesh), Assam (India) and Meghalaya (India) is not just a need it is a necessity for the protection of human lives, food security, and sustainable development in Bangladesh.

For rainfall forecasting, time series models like ARIMA and SARIMA have previously been used by researchers extensively, but they tend to struggle to account for rainfall variability in terms of noises involved in historical data^7–11^. Different time series models have been developed to forecast rainfall in Sylhet, Chittagong, Assam, and Meghalaya, the regions of interest of this research, each of which is specific to their respective climatic patterns^7,12–14^. However, high error rates in forecasting have been reported in these investigations. While machine learning models, such as Support Vector Machines (SVM)^15,16^, Artificial Neural Networks (ANN)^17,18^, and Long Short-Term Memory (LSTM)^19^ have been demonstrated to outperform conventional time series models. Not only in the regions covered by this research work but in the world, as a whole, these ML models outperform the traditional models. Research^20–30^ employed ANN to predict rainfall, and research^31–40^ employed SVM. Research^41^ employed LSTM, Stacked-LSTM, Bidirectional-LSTM Networks, XGBoost, and an ensemble of Gradient Boosting Regressor, Linear Support Vector Regression, and an Extra-trees Regressor have been utilized for the said purpose as well. Research^40,42^, employed Decision tree, Naïve Bayes, K-Nearest Neighbors, and Support Vector Machines, Bayesian Linear Regression (BLR), Boosted Decision Tree Regression (BDTR), Decision Forest Regression (DFR) and Neural Network Regression (NNR). Research^43^ applied Linear Regression, Multi-Layer Perceptron (MLP), Convolutional Neural Network (CNN), Long Short-Term Memory (LSTM), Gated Recurrent Unit (GRU), and Bidirectional Long Short-Term Memory (BLSTM) for rainfall modelling. A number of hybrid models have been proposed in recent years to enhance the accuracy of rainfall forecasting. Research^44^ combined Convolutional Neural Networks (CNN) with Long Short-Term Memory (LSTM) networks in a successful effort to detect spatial and temporal correlations in rainfall data. Research^45^ employed a Neural Network reinforced by Gaussian Random Fuzzy Numbers, and tree models such as Random Forest and Dual Perturb and Combine Tree, in a bid to improve prediction accuracy. Research^46^, however, employed lazy learner approaches such as K-star and instance-based K-nearest neighbors hybridized with Rotation Forest (ROF) model. These methods were chosen for their ability to extract meaningful patterns from past rainfall data and, subsequently, improve the precision and reliability of rainfall predictions.

The intricate relationship between meteorological factors and inherent noise in short-term rainfall records continues to be a concern for refining the precision of rainfall forecasting further. While machine learning (ML) models have been shown to outperform their time series counterparts, the high forecasting errors in the literature accentuate the need for more sophisticated methods. To address these challenges, researchers have explored various hybrid techniques for rainfall forecasting. In particular, the use of advanced signal processing techniques e.g., Empirical Mode Decomposition (EMD), Ensemble Empirical Mode Decomposition (EEMD), Discrete Wavelet Transform (DWT), and Singular Spectrum Analysis (SSA) has proven to be extremely beneficial in feature extraction for ML models^47–52^. Such techniques help break down intricate, non-stationary rainfall data into understandable parts, improving model performance. Although hybrid models are successful, there is a need for further improvement to mitigate the effects of noise and data variability. Addressing these limitations is important not just for use in areas covered by current research but also for worldwide application. Therefore, there is a need to formulate more robust and advanced models to enhance the precision and reliability of rainfall prediction significantly. However, the complex interplay of meteorological variables and the inherent noise in short-term rainfall data demand more powerful and nuanced solutions.

Artificial Neural Networks (ANNs) have been found to be effective tools for non-linear modeling of hydrological behavior^53^^,54^. ANN is one of the most well-known algorithms for advanced tasks that can handle high-level data patterns^55^. Scientists have used ANN in rainfall forecasting to overcome these challenges effectively, increasing performance and precision. Its layered architecture, activation functions, and adjustable weights enable them to learn intricate patterns in challenging datasets^40^. However, the performance of ANNs is critically dependent on the optimal tuning of their weights^56^. An effective weight initialization technique would preferably initialize the weights to maximize the speed of training as well as the performance of the neural network^57^. Historically, optimizations like Gradient Descent and its variants have been employed for this purpose^58^. While appropriate for most applications, these gradient-based methods tend to get stuck in local minima and struggle in the high-dimensional, non-convex topographies of ANN weight spaces^59^. Furthermore, metaheuristics like Genetic Algorithms (GA)^60,61^, Particle Swarm Optimization (PSO)^62^, Ant Colony Optimization (ACO)^63^, and Differential Evolution (DE)^64^, Harmony Search Algorithm^65^, Simulated Annealing (SA)^66^, ant-lion optimization^67^, Bat algorithm (BA)^68^ have been extensively employed in an attempt to circumvent these limitations. These methods enable global search but are slow to converge and computationally expensive. Despite these advances, the efficient and effective optimization of ANN weights for challenging hydrological data, particularly in the context of short-term rain forecasting, remains a daunting task, often requiring innovative approaches to crossing the high-dimensional search space without overfitting. There is an increasing necessity for the development of a more powerful and efficient optimization system to enhance performance and accuracy in finding solutions to complicated problems. Alternatively, existing optimization processes have to be developed further through the inclusion of hybrid methods, which combine several methods to achieve a more accurate optimal solution.

This study addresses the limitations of existing methodologies, including the challenge of addressing noise and complexity in short-term rainfall data, the application of single-step optimization in ANN weight adjustment, and the lack of leverage for recent and robust nature-inspired metaheuristic optimization algorithms. To address these limitations, this study proposes a new dual-step optimization framework for ANN weight optimization that is crucial in guaranteeing high predictability accuracy in complex data rainfall structures. This study utilizes recent nature-inspired metaheuristic algorithms Egrit Swarm Optimization (ESOA), Harris Hawks Optimization (HHO), and Hippopotamus Optimization (HO) that are not extensively applied in rainfall modeling, and well-known optimizers like PSO and GA. Egret Swarm Optimization (ESOA), a metaheuristic optimization algorithm based on the hunting behavior of egrets, was introduced in 2022^69^. Harris Hawks Optimization (HHO), also presented in 2019, is derived from the group behavior and natural surprise pounce hunting tactic of Harris’ hawks in nature^70^. Hippopotamus Optimization (HO), introduced in 2024, draws inspiration from the natural inherent behavior observed among hippopotamuses^71^. These recently developed nature-inspired metaheuristic optimization methods have vast possibilities to optimize the weights of ANN in noisy and complicated rainfall data conditions. This research not only intrigues these newly developed algorithms but also introduces a novel dual-stage optimization framework for the optimization of ANN weights, and it proves the strength and efficacy of such algorithms in improving the performance of the model. The proposed method begins with HHO which efficiently explores the search space for a well-conditioned and converged set of weights and prevents early convergence. Subsequently, in the second step, a supporting optimization (HHO, ESOA, HO, GA and PSO) algorithm adjusts and optimizes these weights to get the best possible convergence. Besides, this paper extends weight optimization by incorporating probabilistic optimization in the form of Bayesian Optimization (BO) into fine-tuning the hyperparameters of the model. This blend method both enhances weight learning and hyperparameter adjustment, offering a stronger and better-performing ANN-based rainfall forecasting technique.

Moreover, this study introduces a novel application of Non-negative Matrix Factorization (NMF) for short-term rainfall data preprocessing and feature extraction. While NMF has been used in various fields, its application to hydrological time series, particularly rainfall forecasting, is not widespread^72,73^. Additionally, NMF has been effectively used in rainfall-runoff modelling, with high accuracy and reliability in illustrating the complex nonlinear relationship between rainfall inputs and runoff responses^74^. NMF’s inherent non-negativity constraint is best suited for rainfall data because of its intrinsic non-negative nature, thereby ensuring physical interpretability and model stability^75^^,76^. Furthermore, this research proposes a novel data-driven component selection method for NMF based on the analysis of reconstruction error plots and their derivative counterparts. This approach is a far cry from traditional heuristic-based methods and offers an objective and stable way of determining the optimal dimensionality reduction.

By coupling this novel dual-step metaheuristic optimization with the application of NMF and its data-driven component selection method, this research provides an integrated and efficient framework for flood-prone area rainfall forecasting like Sylhet, Chittagong, Assam and Meghalaya. This not only surpasses the issues of noise and non-linearity but enhances the robustness and accuracy of ANN-based rainfall forecasting as well. The value of this work lies in being able to provide more accurate and credible rainfall prediction, thereby better flood risk management, disaster readiness, and sustainable development in Bangladesh.

## Theoretical background

### Non-negative matrix factorization

**Fig. 1:**
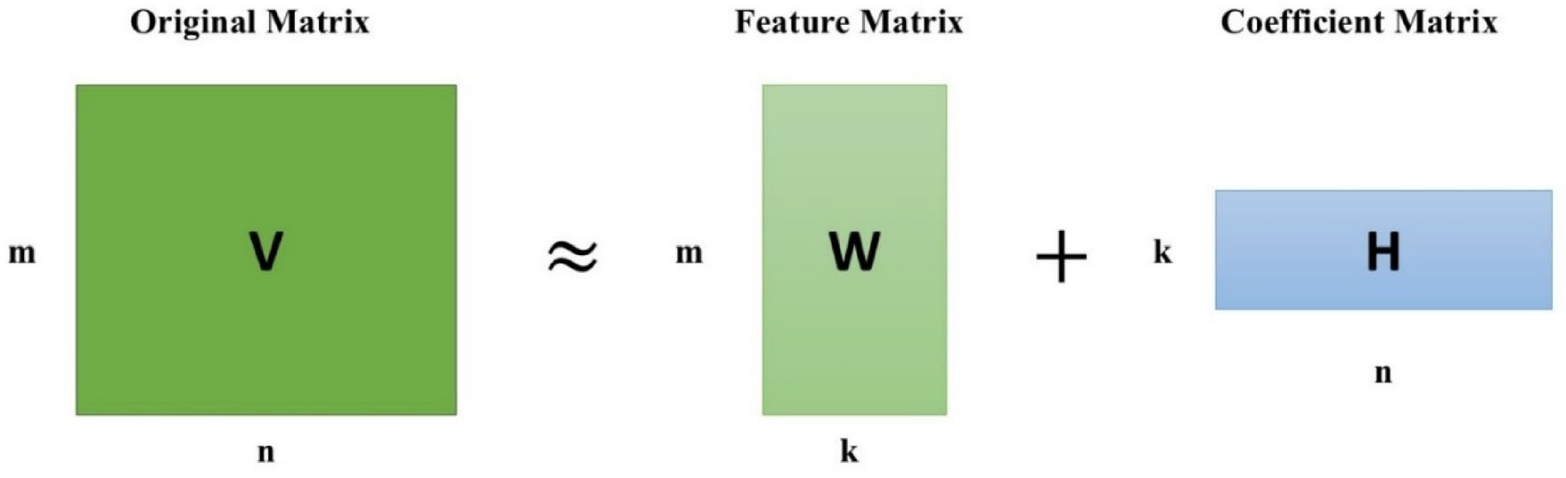
Matrix formation of NMF.

Non-negative Matrix Factorization (NMF) is a dimensionality reduction and matrix factorization technique that factorizes a non-negative matrix V into the product of two lower-rank non-negative matrices: V ≈ WH. Where, V is the original m×n non-negative matrix, W is an m×k non-negative matrix (basis matrix), H is a k×n non-negative matrix (coefficient matrix), k is the reduced rank (chosen to be smaller than both m and n). Figure 1 shows the matrix formation. NMF factorizes V in such a manner that: Columns of W are latent patterns or features of the data. Columns of H contain the weights or contributions of these features in the approximation of V. Mathematically, NMF finds W and H by optimizing the issue (Eq. 1):

1 $$\:{min}_{W,H\:}{\left|\right|V-WH\left|\right|}_{F}^{2}$$

Subject to W, H ≥ 0, where ∣∣⋅∣∣F denotes the Frobenius norm. Due to the non-negativity of matrix entries, the decomposition naturally defaults to an additive, part-based description of the data. In contrast to approaches like Principal Component Analysis (PCA), which allows negative components, NMF focuses on constructive combinations and therefore lends itself to interpretability in real datasets. For instances like rainfall data analysis, W may represent basic factors and H signifies to what degree each factor is involved.

### Artificial neural network

**Fig. 2:**
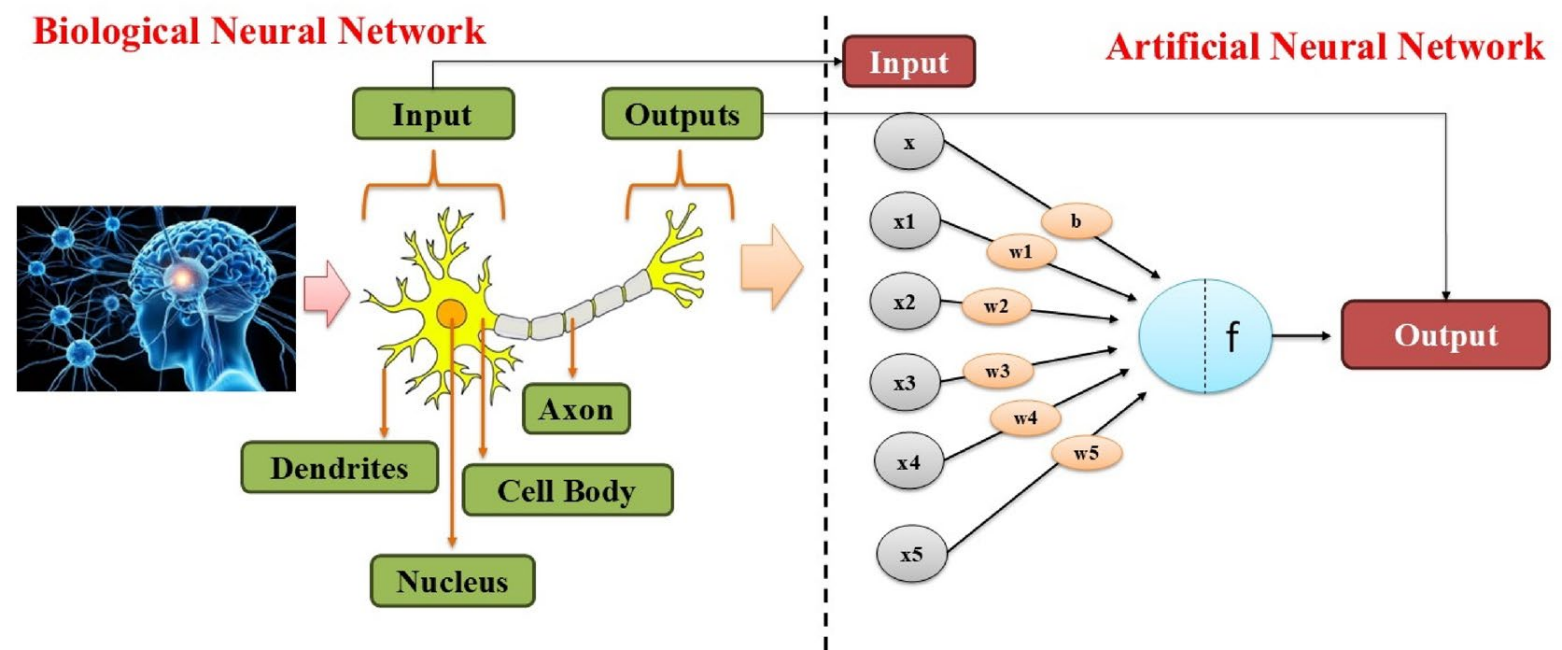
Basic working system of neural network.

Artificial Neural Network (ANN) is computer simulations using the structure and function of living neural networks^6^. ANN consists of processing nodes called neurons, which are networked in layers: an input layer, hidden layers, and an output layer^77^. Figure 2 shows the basic working system of Neural Network. The fundamental mechanism of an ANN is to get input data, process it by weighted connections, apply activation functions, and then generate an output^77^. Mathematically, the output of a neuron in a hidden layer is given by Eq. 2:

where. xi represents input features, wi are the associated weights, b is the bias term and f(.) is the activation function^56^. A single hidden layer was employed in the artificial neural network, (ANN) model, and the number of hidden nodes was determined through iterative experimentation^78^. This study used the activation functions Leaky ReLU, ReLU, tanh, and sigmoid. The behavior of these activation functions is shown in Eqs. (3-6).

2 $$Z = f~\left( {\mathop \sum \limits_{{i = 1}}^{n} w_{i} x_{i} } \right) + b$$

3 $$Sigmoid:{\text{ }}f\left( x \right){\text{ }} = \frac{1}{{1 + ~e^{{ - x}} }}$$

4 $$\text{Tanh} :{\text{ }}f\left( x \right){\text{ }} = \frac{{e^{x} - e^{{ - x}} }}{{e^{x} + e^{{ - x}} }}$$

5 $${\text{ReLU}}:{\text{ f}}\left( {\text{x}} \right){\text{ }} = {\text{ max }}\left( {0,{\text{ x}}} \right)$$

6 $${\text{Leaky ReLU}}:{\text{ f}}\left( {\text{x}} \right){\text{ }} = {\text{ max }}\left( {\alpha {\text{x}},{\text{ x}}} \right),{\text{ where }}\alpha {\text{ is a small constant}}$$

A transfer function, also referred to as an activation function, is a fundamental function that produces an output depending on the net value of the input, which is the result of the weighted sum of the inputs and biases^79^. the final output of the ANN is computed as (Eq. 7):

7 $$Y = f_{{out}} \left( {\mathop \sum \limits_{{j = 1}}^{m} w_{j} Z_{j} + b_{{out}} } \right)$$

Where $\:{Z}_{j}$ are activations from the hidden layer, $\:{w}_{j}$ are weights of the output layer, and $\:{b}_{out}$ is the bias term.

### Metaheuristic optimization

This study employs nature-inspired metaheuristic optimization algorithms for modeling rainfall using artificial neural networks (ANN). It contrasts the performance of newly introduced and popular algorithms, namely Harris Hawks Optimization (HHO), Egret Swarm Optimization Algorithm (ESOA), and Hippopotamus Optimization (HO), with conventional ones, namely Genetic Algorithm (GA) and Particle Swarm Optimization (PSO). There is abundant literature in favor of the effectiveness of GA and PSO for ANN-based prediction issues in a broad spectrum of areas, including but not limited to rainfall prediction^80^.

On the other hand, HHO, HO, and ESOA are recent metaheuristic developments, inspired by the complex behavioral patterns of animals and swarms. These algorithms are targeted at solving highly nonlinear optimization issues and have demonstrated promising results in various applications. Their use in rainfall modeling is largely untested, and hence, their application in the current research forms a new contribution. By including both classical and new optimizers, this research provides a comprehensive assessment of their impact on ANN performance in rainfall prediction. In the interest of maintaining the brevity of the main manuscript, detailed mathematical equations and algorithmic descriptions of HHO, HO, ESOA, GA, and PSO are provided in Appendices A, B, C, D, and E, respectively.

**Fig. 3:**
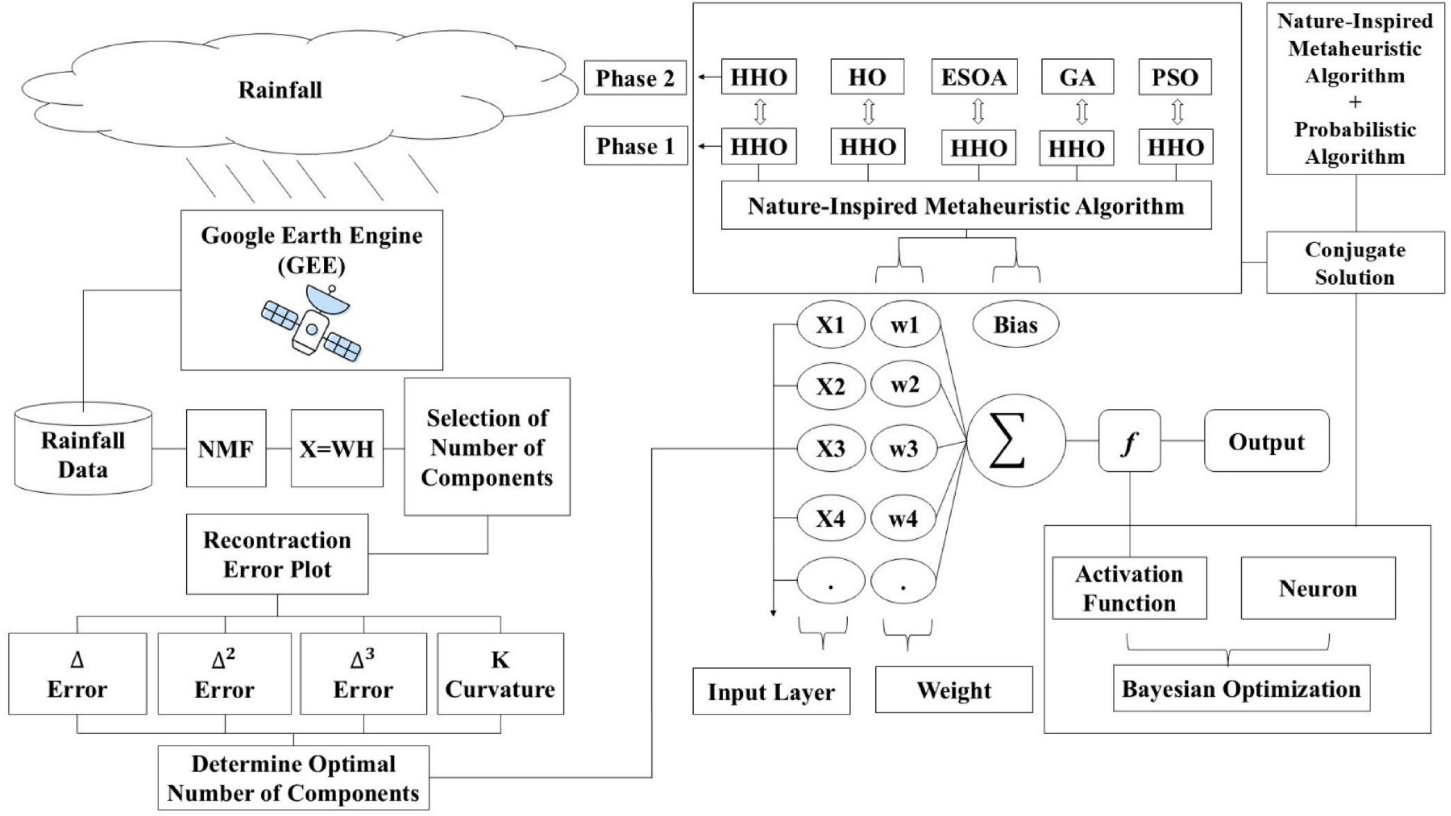
Proposed framework.

## Design and implementation of the proposed method

This study introduces two novel approaches that are aimed at enhancing the performance of predictive models for rainfall data. It initially applies Non-negative Matrix Factorization (NMF) as a data preprocessing technique to rainfall data, which is then provided as input to an Artificial Neural Network (ANN) model. NMF is used to factor out underlying features from rainfall data, thus obtaining dimensionality reduction and improved input feature quality for the ANN. This preprocessing step helps in the improved capture of hidden patterns in the data and thus improves the predictive accuracy of the ANN model.

In the second approach, three newly introduced nature-inspired metaheuristic algorithms such as Harris Hawks Optimization (HHO), Hippopotamus Optimization (HO), and Egret Swarm Optimization Algorithm (ESOA) and two classical algorithms such as Genetic Algorithm (GA) and Particle Swarm Optimization (PSO), are utilized for optimizing the weights of the Artificial Neural Network (ANN) model. In this study, a novel two-stage optimization framework is proposed, wherein the algorithms are combined in hybrid pairs: HHO-HHO, HHO-HO, HHO-ESOA, HHO-GA, and HHO-PSO. The efficiency of these hybrid two-step optimization methods is compared to that of conventional single-step optimization by the respective algorithms (HHO, HO, ESOA, GA, and PSO). The paradigm, not only takes advantage of each optimization technique in isolation but synergistically benefits from a combined approach for achieving superior results in fine-tuning the parameters of the model. The synergistic combination of NMF preprocessing and two-step optimization produces a robust platform for accurate rainfall forecasting, one of the significant advances in predictive modeling. The two major sections are divided into the preprocessing phase and the optimization phase, which represent the primary objective and novelty of this research. The overall methodology is presented in in Fig. 3.

### Preprocessing phase

In the first step, Non-Negative Matrix Factorization (NMF) is applied for preprocessing the data before Artificial Neural Network (ANN) training. NMF is a technique of matrix factorization that breaks down a given non-negative matrix X, rainfall data, into two non-negative matrices: W (Basis Matrix) and H (Coefficient Matrix). It decomposes iteratively following a multiplicative update rule. The selection of the number of NMF components is a critical choice because it directly influences model accuracy, training time, and the ability to generalize. An inappropriate decision may lead to underfitting if too few features are chosen, resulting in a loss of useful information, or overfitting if too many features are picked, incorporating irrelevant or noisy features. Since ANN training requires an optimized feature space, a wrong choice of features aggravates model complexity by necessitating additional layers for feature extraction, affecting weight initialization and convergence rate in ANN training. To determine the optimal number of components, a systematic approach is taken in which the number of components is changed and the reconstruction error is evaluated. The aim is to determine where more components do not yield much more improvement in reconstruction accuracy to avoid underfitting and overfitting.

To solve this, the reconstruction error changes are inspected using derivative-based and curvature-based methods. The first derivative ($\:\varDelta\:$ Error) measures the rate of diminishing error with the increase in the number of components, and the second derivative ($\:{\varDelta\:}^{2}$ Error) is the acceleration of diminishing error. The third derivative ($\:{\varDelta\:}^{3}$ Error) provides information about the stability of the second derivative, and the curvature analysis (K) evaluates the bending of the error function to find the greatest improvement in the reconstruction quality. By ascertaining the number of components where the second derivative is maximized, the third derivative peaks, or the curvature is highest, an optimal trade-off is achieved between feature representation and computational complexity. This ensures that the preserved components capture the salient patterns in the data without including redundancy or noise. The low-dimensional representation thus obtained is then used for ANN training, leading to improved model performance and increased generalization capacity.

### Optimization phase

In this study, a two-stage hybrid optimization methodology is presented to enhance the accuracy and efficiency of Artificial Neural Network (ANN) training. The initial stage is used to optimize the ANN architecture, and the second stage applies state-of-the-art hybrid algorithms to optimize the network weights. By combining Bayesian Optimization (BO) to set architecture and a range of robust hybrid optimization techniques to tune weights, the study aims to improve the overall performance of the ANN so that it can converge more quickly with reduced error when trained.

#### Stage 1: Bayesian optimization for architecture configuration

The initial step of the optimization process employs Bayesian Optimization (BO) for the optimal design of the ANN structure (Appendix F). In this scenario, BO is employed for finding two very crucial details in the network environment such as the transfer function and number of hidden neurons. The activation function in each layer in the ANN, i.e., ‘relu’, ‘sigmoid’, ‘tanh’, and ‘leakyrelu’. BO helps in the identification of the best-fitted activation functions. The optimal number of hidden neurons of the ANN, which plays a significant role in determining the capacity and complexity of the model, is optimized using BO. The method looks for various numbers of hidden layers to find the best depth to attain the best performance. Bayesian Optimization is particularly well positioned for this task since it has the capability of considering the probabilistic distribution of performance across different configurations. Probabilistic character allows the optimization process to reflect on uncertainty and aggressively search for the architecture minimizing the validation error (Mean Squared Error or MSE). Utilizing BO ensures the network architecture is selected in an efficient computational, data-driven manner to avoid overfitting or underfitting.

#### Stage 2: weight tuning using hybrid dual stage algorithms

Once the architecture has been optimized in the initial stage, the second phase focuses on fine-tuning the ANN weights using a combination of Harris Hawks Optimization (HHO) and other leading-edge optimization techniques (Appendix A). Hybrid algorithms are used to improve training efficiency and performance through optimal weights for quicker convergence and fewer total errors during training. The overall methodology is illustrated in Fig. 3 as well, and the Pseudocode is provided.

##### Hybrid HHO-PSO (Harris Hawks optimization and particle swarm optimization)

In the HHO-PSO hybrid method, the process begins with HHO conducting a global search for optimal ANN weights in the entire solution space. The HHO algorithm is tasked with searching the weight space widely, identifying regions that are most likely to contain optimal solutions. After potential regions are identified, the Particle Swarm Optimization (PSO) algorithm is employed to locally optimize these solutions (Appendix E). PSO updates the particles’ positions with their individual best-known position and global best-known position discovered by the swarm. The combination of HHO’s global exploration and PSO’s local enhancement ensures strong convergence towards an optimal solution, improving the ANN’s training performance.

*First-stage HHO: global exploration of optimal regions*.

The HHO algorithm is used during the first stage to perform a global exploration of the whole weight space of the ANN. This stage aims to examine different regions and identify regions in which optimal weight settings could lie. HHO mimics the cooperative hunting strategy of Harris hawks with dynamic exploration mechanisms such as:

- Levy flight-based search: The hawks explore the solution space extensively, evading premature convergence by taking huge random leaps.
- Surrounding and preying on the opponent: Solutions get modified based on measurements of fitness, and variable strategies are utilized to stay adaptable.
- Escaping local minima: Using dynamic adjustment in exploration parameters, the initial-stage HHO ensures that the search is over a wide and diverse set of solutions.

After the completion of this phase, the HHO algorithm has shortlisted the most promising regions where optimal ANN weights will be obtained.

*Second-stage PSO: local optimization*.

Once high-potential areas of the search space have been identified by the HHO algorithm, the PSO algorithm performs local optimization and fine-tuning of the selected solutions. It enhances convergence with fine-tuning of weights within the recognized areas. Critical PSO mechanisms of this phase are:

- Particle-Based Search: Each solution is depicted as a particle, which moves across the search space based on the best-known positions.
- Individual and Global Best Updates: Each particle updates its position based on Personal Best Position and Global Best Position.
- Velocity and Position Updates: The movement of particles is controlled using velocity updates, which produce a localized adaptation and gentle convergence.

##### Hybrid HHO-HHO (Harris Hawks optimization and Harris Hawks optimization)

The HHO-HHO hybrid makes use of two instances of the HHO algorithm. In this setup, the first HHO algorithm globally searches the solution space to determine the areas where the potentially optimal ANN weights exist. The second HHO algorithm then fine-tunes these discovered solutions through a local search. This two-stage procedure provides enhanced exploration and exploitation phases in the optimization process. By applying HHO in two stages, the method increases robustness and flexibility to complex search spaces with enhanced performance and accuracy.

*First-stage HHO: local exploitation*.

In the first phase, the HHO algorithm performs a global search across the whole solution space (Appendix A). The prime aim of this phase is to find the potentially good regions in which the optimal ANN weights would be likely located. The HHO algorithm imitates the cooperative hunting behavior of Harris hawks using different strategies such as:

- Exploration phase: The hawks randomly search the search space based on patterns of Levy flights to avoid premature convergence.
- Surrounding prey: The algorithm estimates diverse solutions at each stage, changing their positions based on the solution quality.
- Diverse searching strategies: The search is dynamically diversified across different movement patterns (e.g., soft and hard besiege strategies) for enhancing adaptability.

After this stage, the initial HHO algorithm effectively narrows down the potential regions where better-performing ANN weight configurations are found.

*Second-stage HHO: local exploitation*.

Once the initial HHO has identified potential areas, the second HHO algorithm performs a local search within the more specific areas. The weight values chosen are further refined through finer exploitation methods during this stage, thus better convergence towards the optimal point is guaranteed. The key steps are:

- Focused adjustment: Instead of investigating the entire solution space, this phase operates in the areas found in the first phase, producing faster and better optimization.
- Exploitation phase: The second HHO incidence optimizes solutions so that adaptive dynamic parameters are employed to control the movement of the hawks based on the fitness landscape.
- Avoiding local minima: Through adaptive switching between different escaping and besieging strategies, the algorithm avoids stagnation and improves convergence reliability.

##### Hybrid HHO-HO (Harris Hawks optimization and hippopotamus optimization)

The HHO-HO hybrid method combines HHO with the Hippopotamus Optimization (HO) algorithm (Appendix B). In the hybridization, HHO conducts the global search for promising regions of the weight space, and HO is employed to locally refine these solutions. HO, inspired by hippopotamuses’ territorial and foraging behavior, enhances the algorithm’s ability to explore solution spaces more thoroughly. By combining the global search of HHO with the local exploitation of HO, the hybrid method improves the efficiency and effectiveness of the optimization process with faster convergence to the optimal solution.

*First-stage HHO: global search for promising regions*.

Optimization begins with HHO, the problem of searching the ANN weight space globally for promising regions. Inspired by the distributed hunting behavior of Harris hawks, HHO uses adaptive search strategies to find promising regions:

- Exploration stage: During this stage, HHO employs Levy flight-based random flight, diversifying the search while avoiding premature convergence.
- Surrounding and besieging prey: Several adaptive strategies, such as soft/hard siege mechanisms, provide robust search coverage.
- Adaptive search mechanism: HHO dynamically oscillates between exploitation and exploration based on the fitness landscape. After this phase, HHO has identified high-potential areas in the solution space where the optimal ANN weight configurations are likely to be discovered.

*Second-stage HO: local refinement and exploitation*.

Once HHO has identified potential regions, the HO algorithm is applied to optimize the solutions found. HO is inspired by hippopotamus foraging and territoriality, which is the cause of an effective local search strategy:

- Local exploration based on social behavior: Hippos explore in their territory, a focused improvement of solutions. This mechanism ensures step-by-step but precise modifications in the ANN weight space.
- Self-adaptation strategy: Relative to solely random search algorithms, HO dynamically modifies movement step size based on optimization advancement. This allows HO to enhance the exploitation of near-optimal solutions and enhance the convergence rate.
- Territorial dominance for optimal solution selection: Solutions are updated iteratively about their relative fitness ranking among a population. This method facilitates the removal of suboptimal weights, leading to improved ANN performance.

##### Hybrid HHO-ESOA (Harris Hawks optimization and Egret swarm optimization algorithm)

The HHO-ESOA hybrid method combines the global search capability of HHO with the swarm intelligence of the Egret Swarm Optimization Algorithm (ESOA) (Appendix C). Here, HHO performs the global search for the solution space and finds promising regions for ANN weights. After a global search, ESOA optimizes these solutions with cooperative behavior inspired by that of egret birds, which cooperate to achieve their goals. The cooperative search improves the accuracy of local search and leads to more accurate solutions. The combination of HHO’s global search and ESOA’s local tuning improves the convergence and optimization of the weights of the ANN.

*First-stage HHO: global search for potential regions*.

The optimization process begins with HHO, which performs a wide search of the ANN weight space. The HHO algorithm is inspired by the hunting style of Harris hawks, wherein cooperative hunting strategies are used to locate prey efficiently. Main Mechanisms of HHO during the Global Search Stage:

- Exploration using levy flight movements: HHO explores the solution space randomly first to avoid premature convergence.
- Adaptive switching between phases: HHO switches between different search strategies, such as soft and hard besiege mechanisms, dynamically based on fitness evaluations.
- Identification of high-potential regions: HHO narrows down the search space after a few iterations, pinpointing the high-potential regions where the optimal ANN weights might be located.

HHO has effectively performed a global scan at the end of this phase, identifying the regions in the search space where refinement is needed.

*Second-stage ESOA: cooperative local optimization*.

Once HHO has identified the high-potential regions, the ESOA algorithm is tasked with optimizing and fine-tuning these solutions. ESOA is inspired by the cooperative foraging behavior of egret birds, which work together to locate food efficiently. Key Mechanisms of ESOA during the Local Optimization Phase:

- Cooperative search behavior: ESOA employs group-level intelligence wherein egret-inspired agents plan and coordinate their movement to enhance the quality of the solution.
- Leader-follower dynamics: The best solutions prompt others to shun stagnation and enable perpetual enhancement.
- Dynamic step size adjustment: ESOA adjusts movement strategies by using feedback from fitness, which causes fine-tuning ANN weights accurately.

By integrating cooperation-based intelligence into the optimization process, ESOA avails greater precision in ANN weight optimization, leading to a stronger and more efficient model.

##### Hybrid HHO-GA (Harris Hawks optimization and genetic Algorithm)

In the HHO-GA hybrid approach, HHO performs the global search to identify potential regions for the optimal ANN weights. Once these regions are identified, the Genetic Algorithm (GA) is used to further refine the solutions (Appendix D). GA evolves and searches the population of solutions using evolutionary principles such as crossover, mutation, and selection. The genetic operators introduce diversity into the search process to avoid premature convergence and achieve robust optimization. The interaction between HHO’s global search and GA’s local search accelerates convergence and optimizes the ANN’s weight configuration.

*Initial-stage HHO: global search for potential ANN weights*.

Optimization starts with HHO, which efficiently searches the search space to locate regions of high promise for the best ANN weights. Inspired by the cooperative hunting strategy of Harris hawks, HHO employs adaptive search mechanisms:

Main Mechanisms of HHO in Global Search:

- Exploration using adaptive movements: Employs Levy flight-based randomness to prevent stagnation in local minima.
- Dynamic search strategy: Alternates between soft and hard besiege phases, mimicking real hunting tactics.
- Identification of high-fitness areas: Targets fruitful areas where the best solutions are likely to be.

By this phase, HHO has discovered the most promising ANN weight areas but requires further fine-tuning for higher accuracy.

*Second-stage GA: evolutionary refinement of solutions*.

After HHO has minimized the search space, the Genetic Algorithm (GA) is employed to optimize and fine-tune the solutions further. GA follows natural selection processes, improving solutions over generations. Primary Mechanisms of GA in Local Optimization:

- Crossover (recombination): Combines genetic information from two parent solutions to generate improved offspring solutions.
- Mutation: Introduces random changes in candidate solutions to maintain diversity and avoid premature convergence.
- Selection (survival of the fittest): Chooses the best-performing solutions of the next generation, accelerating convergence towards an optimal ANN weight configuration.

GA’s preservation of diversity ensures that the optimization process will never be trapped in poor local minima, increasing overall robustness.

The hybrid optimization techniques function iteratively, alternating between global exploration and local exploitation. HHO (or some other global search technique) does an initial general search of the weight space to discover regions with possibly optimal ANN weights. Once these regions are discovered, a secondary optimization technique such as HO, HHO, PSO, and GA is used to search the solutions locally for optimization. This balance between exploitation and exploration ensures that the optimization process explores the solution space extensively but converges rapidly to an optimal solution.

### Optimal model hypermeters range

Another crucial aspect in the optimization process is the selection of appropriate hyperparameter ranges, as they play a major role in determining the overall performance of the model. In the present study, the optimization is carried out using various optimizations, where several key parameters must be specified with care. These are the number of hawks, the top number of iterations, the boundary weight constraints, the transfer range function, and the hidden range neuron in the model. The selection of these hyperparameters will directly influence the ability of the model to learn and generalize, so it is crucial to specify a well-defined range for each parameter. However, specifying such ranges has its constraints since too broad a range or too tight a range can lead to poor performance or convergence issues. In this, the size of the population of hawks in the HHO algorithm is selected as 50 and the maximum number of iterations is also selected as 50. In the case of activation functions, four common transfer functions ReLU, sigmoid, tanh, and leaky ReLU are selected to test the performance of each of them for model training. Further, the neurons in the hidden layer are selected between 1 and 30 for selecting the optimal network setting. By accurately tuning these hyperparameters, the model aims to achieve improved predictive accuracy and stability as well as fix potential problems related to the optimization process.

### Pseudocode of the proposed method


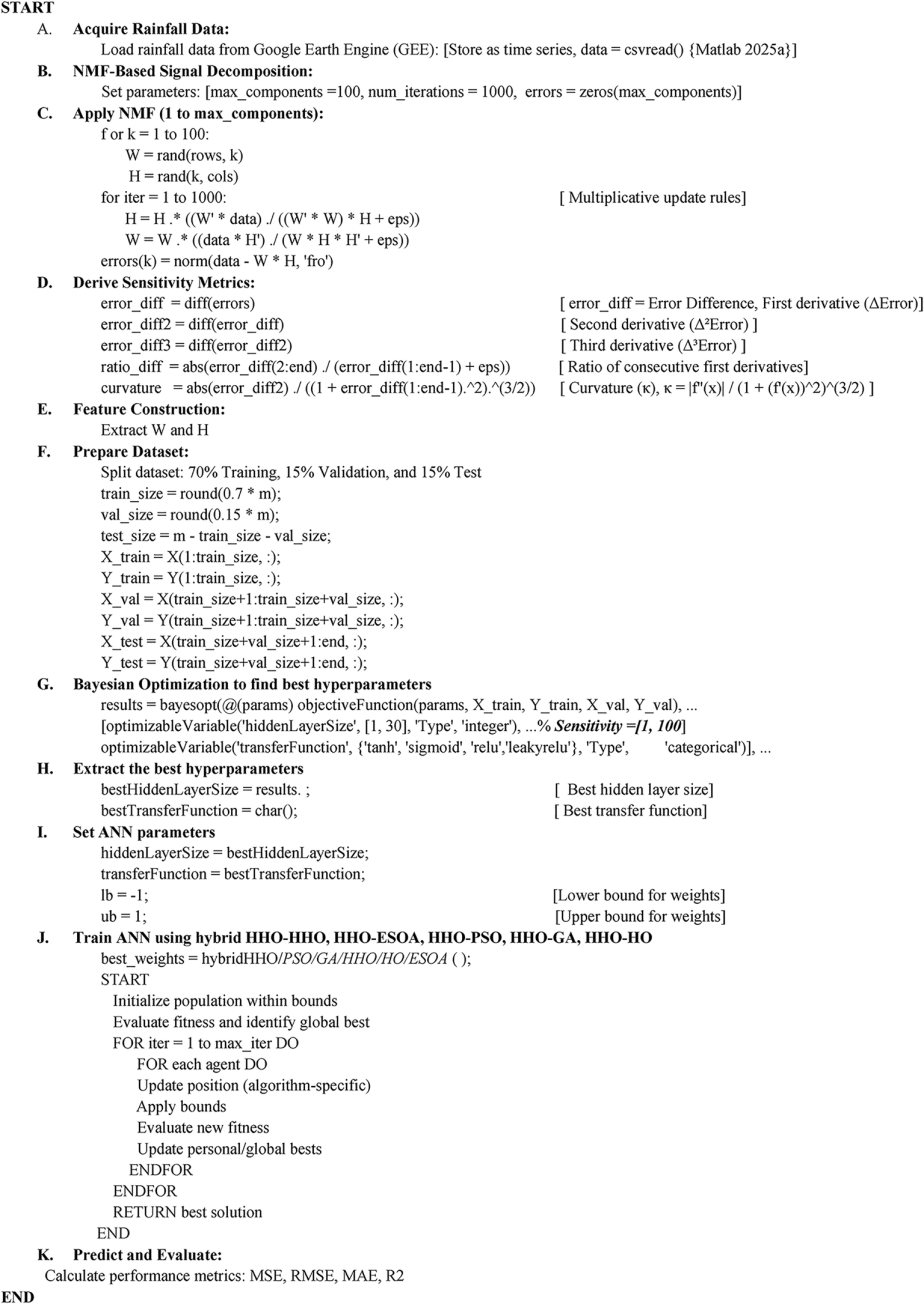


**Fig. 4:**
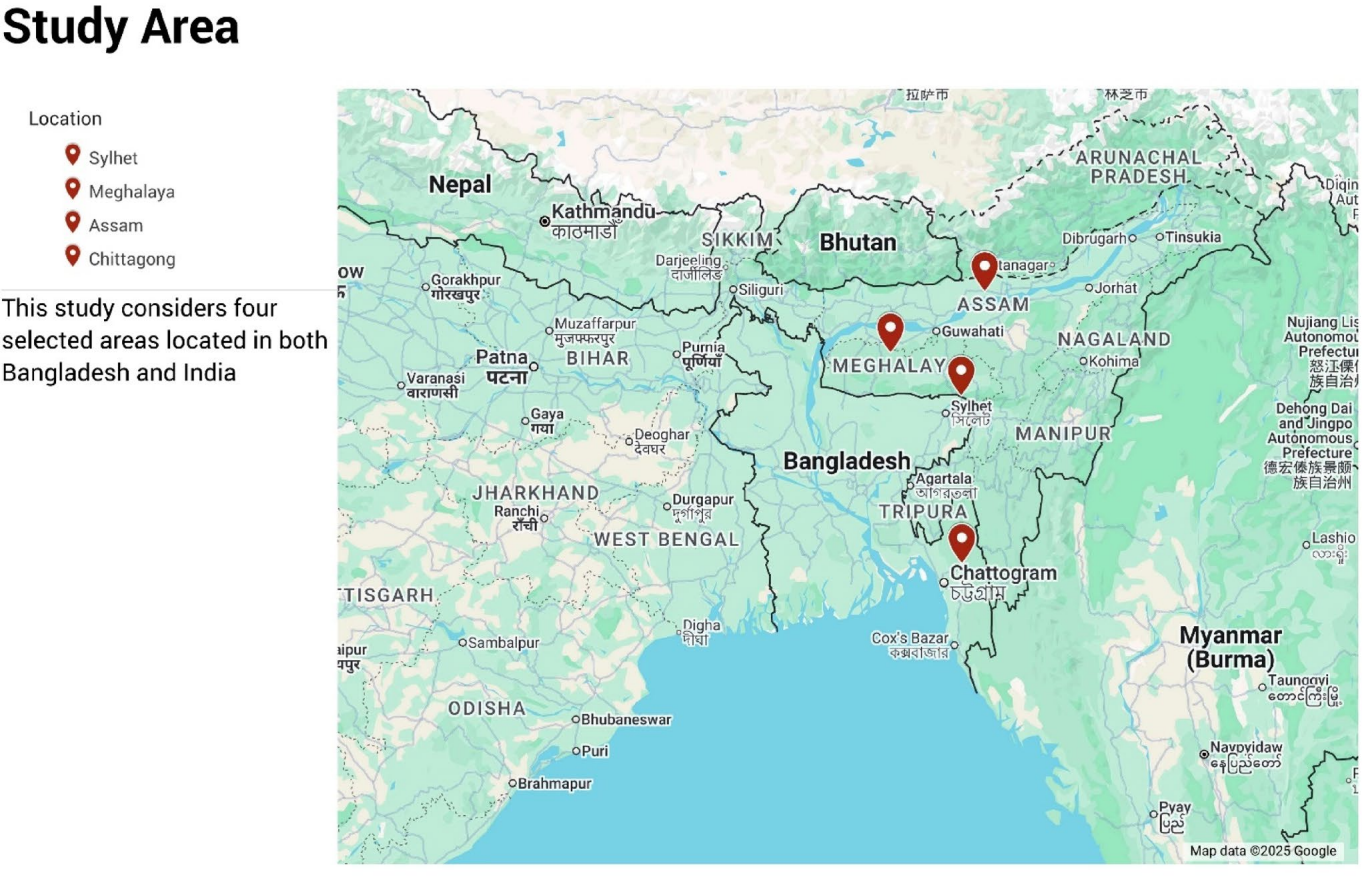
Study area map.

## Study area and data

Four major heavy precipitation areas have been selected in this study: Bangladesh’s Chittagong and Sylhet and India’s Assam and Meghalaya (Fig. 4). These regions contribute significantly to flooding annually in Bangladesh. Flooding occurs annually, and it primarily occurs due to intense rainfall over these catchment areas. Heavy monsoon rains fill the rivers within a very short time, which creates widespread inundation all over Bangladesh. Each year, torrential floods cut short livelihoods and infrastructure and gravely imperil agriculture, public health, and regional ecosystems. It is therefore vital to have accurate rainfall modeling in these regions for the improvement of flood management procedures in Bangladesh.

**Fig. 5:**
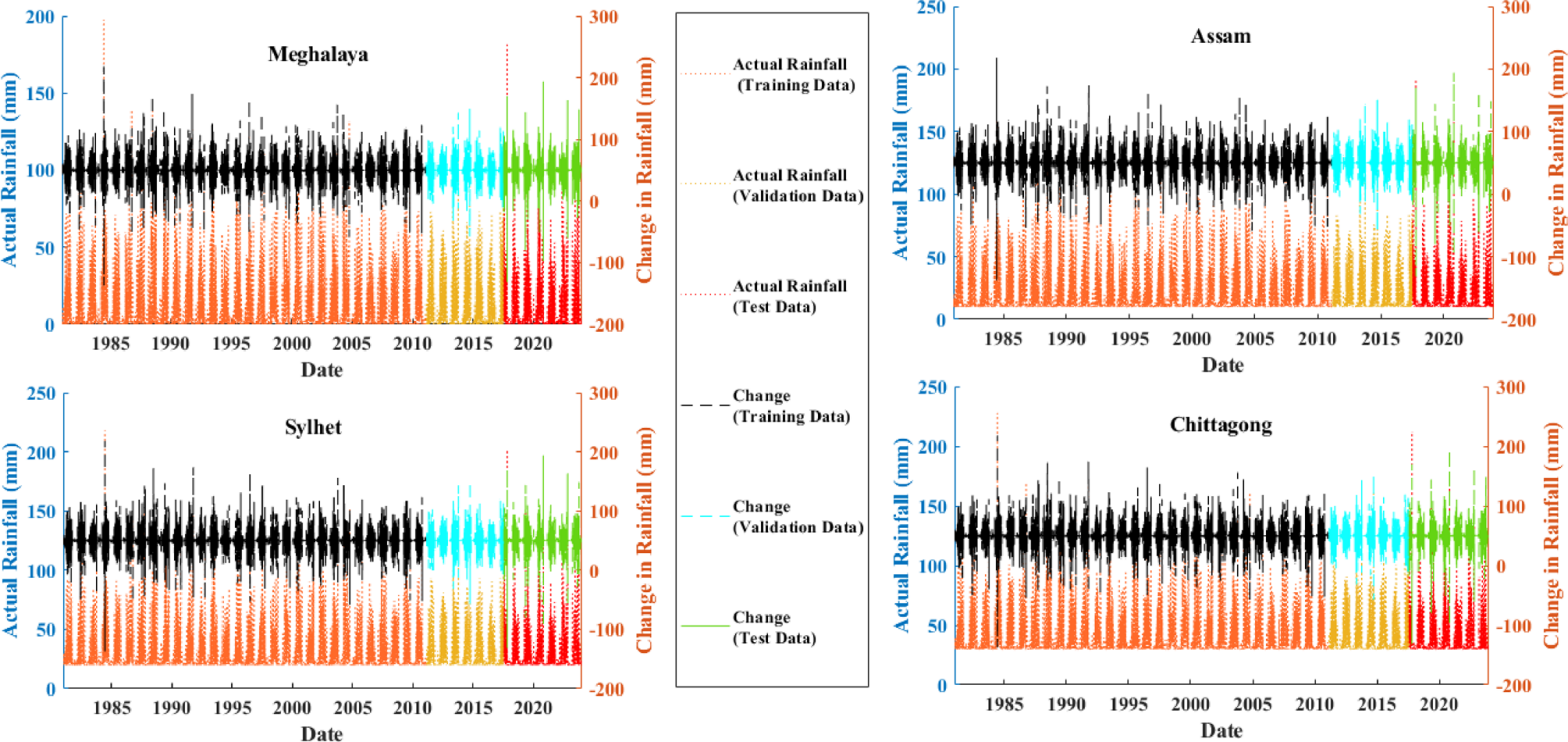
Time series plot of rainfall.

The data required for developing the model on rainfall prediction was collected using Google Earth Engine (GEE)^81^. GEE is a newly developed cloud-based geospatial analysis platform that allows users to visualize and analyze satellite images of the planet. This study uses a dataset comprising 43 years of daily rainfall data, ranging from 1981 to 2023. Such a long-term dataset will catch both the short- and long-term rainfall trends^82^^,83^^,84^. While developing a machine learning (ML) model, data split for training, validation, and testing is a critical process. Based on available research, it has been predominantly noted that the employment of the 70:15:15 split provides an effective and well-structured approach. The said split allows sufficient data for learning (70% training), hyperparameter tweaking and fine-tuning (15% validation), and final performance evaluation (15% testing), leading to stable and consistent predictions^85,86^. The rainfall data gathered was appropriately preprocessed to determine its accuracy and consistency. Missing values were handled either by interpolating or replacing them with the mean of surrounding observations. The map of the study area shown in Fig. 4 was generated using Google Maps (https://maps.google.com/). Historical rainfall records for the study area are shown in Fig. 5.

## Model performance evaluation

Several key statistical metrics exist that can help in measuring accuracy and efficiency for the predictive models, each providing different insights. Table 1 shows all evaluation matrices. These key metrics in this research include Mean Squared Error (MSE), Root Mean Squared Error (RMSE), Mean Absolute Error (MAE), and the Coefficient of Determination (R^2). Mean Squared.

**Table 1:** Evaluation matrix.

Evaluation matrix	Equation	Description
Mean square error (MSE)	$MSE = \left( {\frac{1}{n}} \right)\sum {\left( {y_{i} - \bar{y}} \right)} ^{2}$ (8)	Where n is the number of data points, y_i_ is the i^th^ actual value, ȳ is the mean of the actual values
Root mean square error (RMSE)	$RMSE=\:\sqrt{\left(\:\frac{1}{n}\:\right)\:\sum\:({y}_{i}\:-\:\stackrel{-}{y})2}$ (9)	Where n is the number of data points, y_i_ is the i^th^ actual value, ȳ is the mean of the actual values
Mean absolute error (MAE)	$MAE = \left( {~\frac{1}{n}~} \right)~\sum (y_{i} - \bar{y})$ (10)	Where n is the number of data points, y_i_ is the i^th^ actual value, ȳ is the mean of the actual values
Coefficient of determination (R^2)	$R^{2} = 1 - \left( {\frac{{\sum \left( {y_{i} - \bar{y}~} \right)^{2} }}{{\sum \left( {y_{i} - \hat{y}~} \right)^{2} }}} \right)$ (11)	Where y_i_ is the i^th^ actual value, ȳ is the mean of the actual values, ŷ is the predicted value.

Error (MSE) computes the average of squared differences between actual and predicted values, as represented by Eq. 8. MSE punishes larger errors more than smaller ones and is therefore outlier-sensitive. Smaller MSE indicates a better model. Root Mean Squared Error (RMSE) is the square root of MSE, as represented by Eq. 9, which has the same unit as the target variable and is, therefore, easier to interpret. It is also susceptible to big errors and is usually utilized in evaluating regression models. Mean Absolute Error (MAE) approximates the mean absolute differences between actual and predicted values, and it is denoted as Eq. 10. MAE doesn’t square the errors as MSE and RMSE do, and therefore it isn’t as much influenced by outliers. Lower MAE indicates greater predictive accuracy. The coefficient of Determination R^2 estimates how well the model fits to describe the variance in data and is defined as Eq. 11. It takes values between 0 and 1, where higher values indicate a better fit, although it may turn out to be negative if the model is poorer than predicting the mean. In general, MSE and RMSE put more emphasis on larger errors, MAE provides a straightforward average error metric, and R^2 quantifies the model’s explanatory power.

**Fig. 6:**
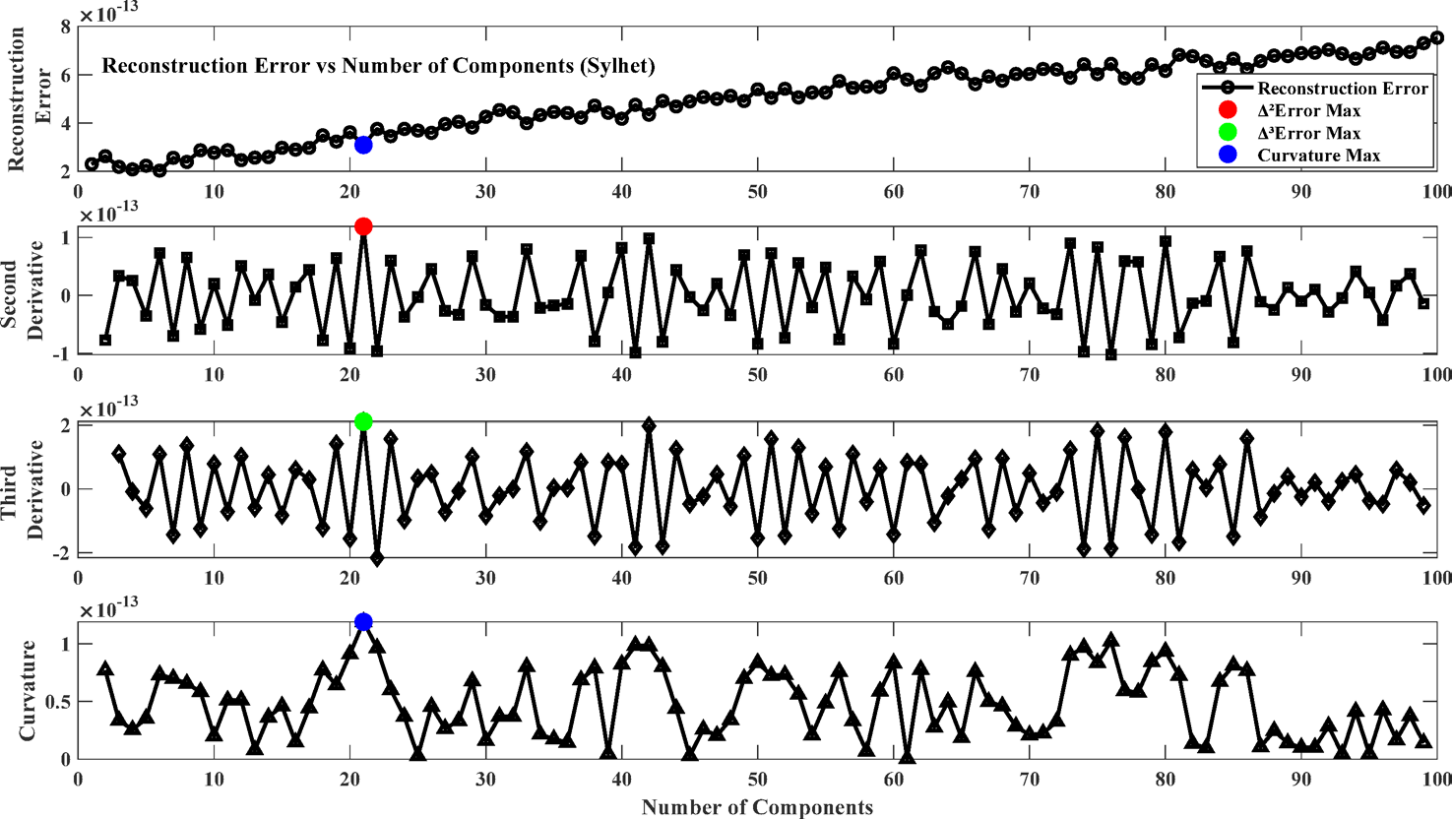
Reconstruction error plot and its derivatives (Sylhet).

**Fig. 7:**
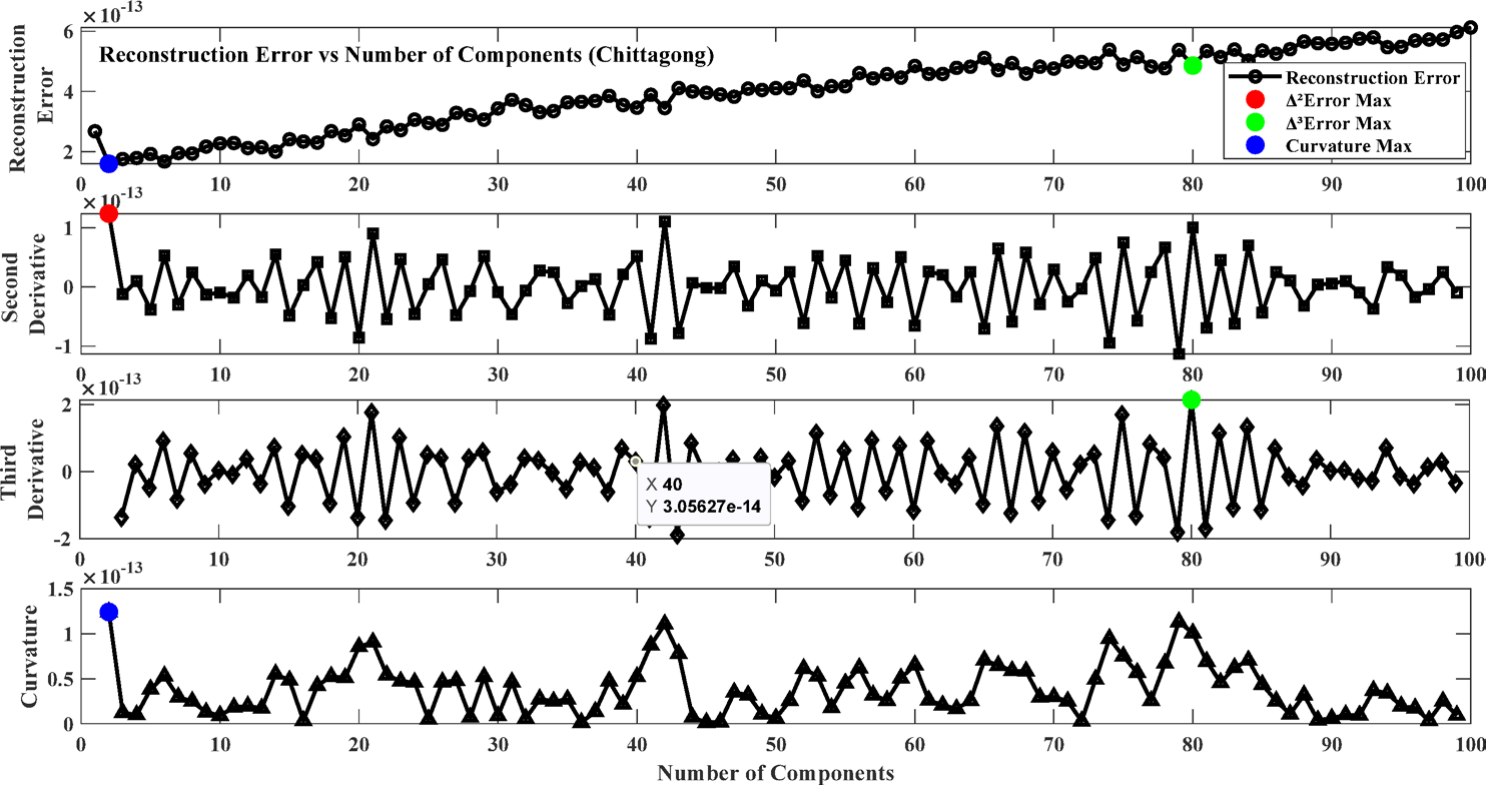
Reconstruction error plot and its derivatives (Chittagong).

**Fig. 8:**
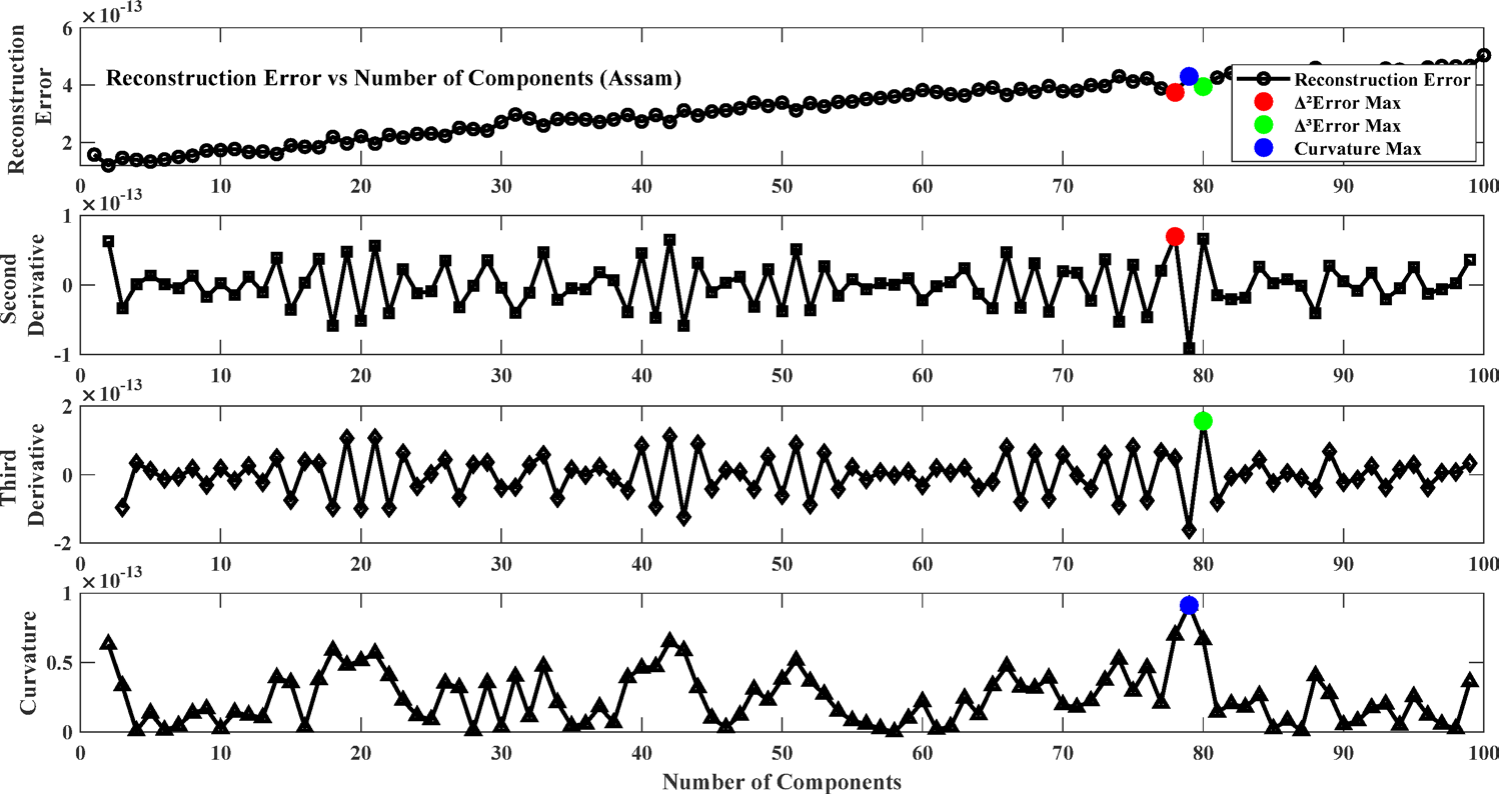
Reconstruction error plot and its derivatives (Assam).

**Fig. 9:**
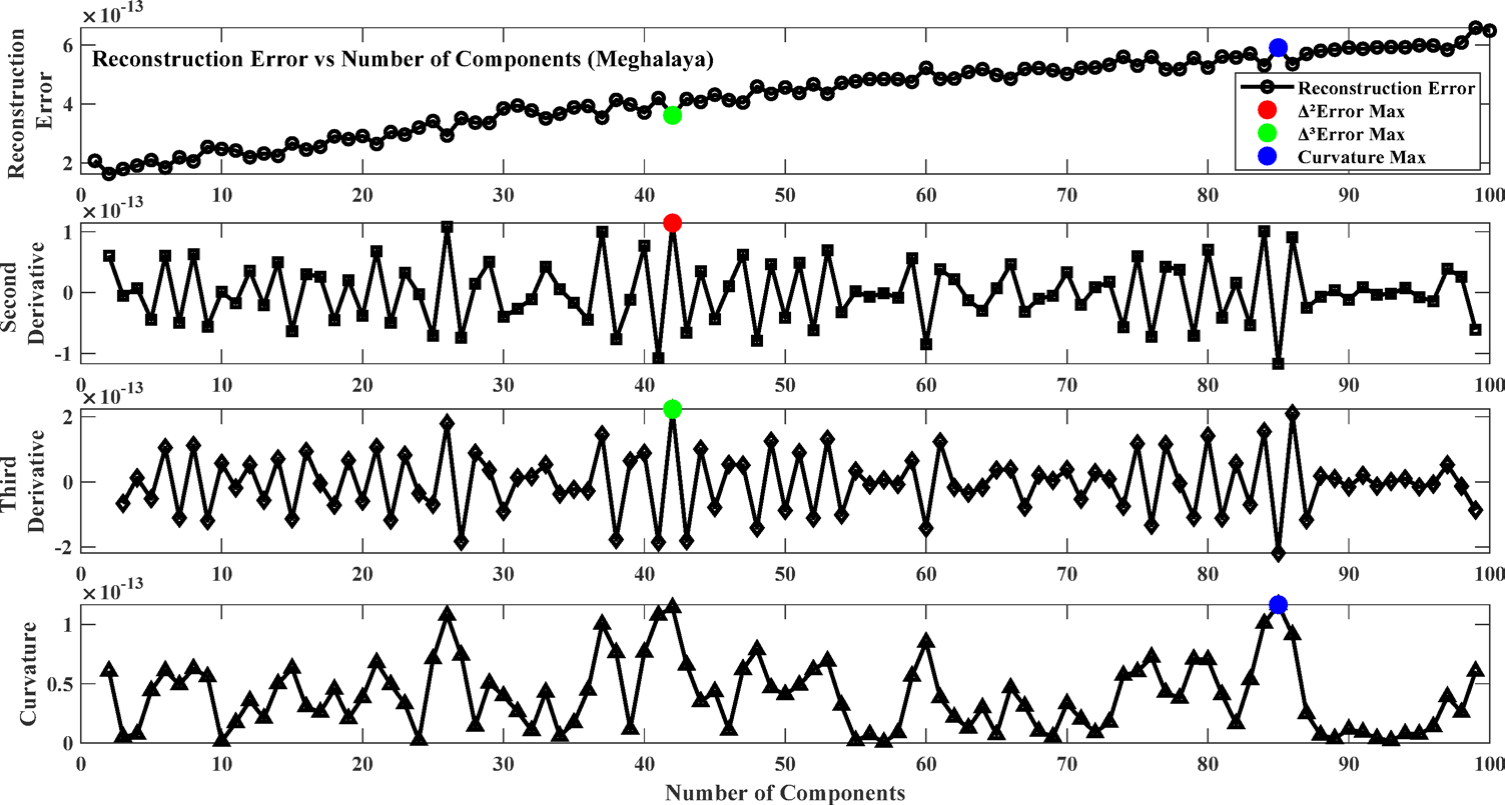
Reconstruction error plot and its derivatives (Meghalaya).

## Result and discussion

This research presents a rainfall prediction model for Chittagong, Sylhet, Assam and Meghalaya. Heavy rainfall at these locations has been the primary driver of flooding in Bangladesh every year. In this research, Artificial Neural Networks (ANN) are applied to predict rainfall with a focus on enhancing ANN training through the implementation of Non-negative Matrix Factorization (NMF). Besides, this work integrates newly proposed nature-inspired metaheuristic optimization algorithms like Harris Hawks Optimization (HHO), Hippopotamus Optimization (HO), and Egret Swarm Optimization Algorithm (ESOA), and well-known optimizers like Genetic Algorithm (GA) and Particle Swarm Optimization (PSO) in optimizing the ANN weights and biases. This paper also introduces a new dual-step approach through the combination of algorithms like HHO-HHO, HHO-HO, HHO-ESOA, HHO-GA, and HHO-PSO to produce better prediction capabilities. Moreover, a probabilistic optimization technique such as Bayesian Optimization is employed to optimize the optimal ANN transfer function and number of hidden layers.

### Result of model training phase

The first step involves training the Neural Network using Non-negative Matrix Factorization (NMF) where the NMF number of components is optimized by selecting the optimal number of components from the reconstruction error plot. The objective is to select the point beyond which more components give minor improvements in reconstruction accuracy to avoid underfitting and overfitting. To achieve this, the reconstruction error changes are analyzed based on derivative-based and curvature-based methods. The first derivative (∆Error) measures the rate at which the error drops with an increasing number of components, and the second derivative (∆²Error) measures the acceleration of this error reduction. The third derivative (∆³Error) measures the stability of the second derivative, and curvature analysis (K) evaluates the bending of the error function to identify the peak improvement in reconstruction quality. Figures 6, 7, 8 and 9 plot the error and its derivatives, and the selection of the training component (W) is done based on these analyses. Table 2 shows the optimal number of components based on the Figs. 6, 7, 8 and 9.

In this study, the second derivative of the error (Max Δ²Error) was used as the criterion for selecting the optimal number of components in the Non-Negative Matrix Factorization (NMF) method. The second derivative can correctly identify the “elbow point,” where the rate of reduction in error plateaus, giving a good trade-off between model complexity and accuracy^87^. As opposed to the third derivative (Max Δ³Error) or curvature (Max Curvature), which may be too sensitive to minor variations and noise, the second derivative is a more interpretable and stable choice criterion^88^. Component selection based on the second derivative also avoids overfitting by retaining only the meaningful components and avoiding unnecessary computational complexity^89^.

**Table 2:** Optimum number of components.

Location	Δ²Error	Δ³Error	Max curvature
Sylhet	21	21	21
Chittagong	2	80	2
Assam	78	80	79
Meghalaya	42	42	85

### Result declaration of the optimization phase

In the optimization phase of the Artificial Neural Network (ANN), Bayesian Optimization (BO) is utilized to start with the optimization of the best hyperparameters, e.g., the training function and structure of the hidden layers. The results of the Bayesian Optimization for each station are shown in Table-3. Then the weight optimization of the ANN model is carried out. The model.

is then subdivided into different single-objective optimization techniques, which are referred to as HHO, ESOA, HO, GA, and PSO. All of these techniques optimize a single parameter or objective independently to enhance the performance of the ANN. In.

addition, the study analyzes dual-step optimization models that integrate two different optimization techniques step by step. These dual-step models introduce combinations such as HHO-HHO, HHO-ESOA, HHO-HO, HHO-GA, and HHO-PSO. By applying these optimization techniques step by step, the dual-step models attempt to elevate the entire optimization process and model performance to an even greater level.

#### Results obtained for the Sylhet region

**Fig. 10:**
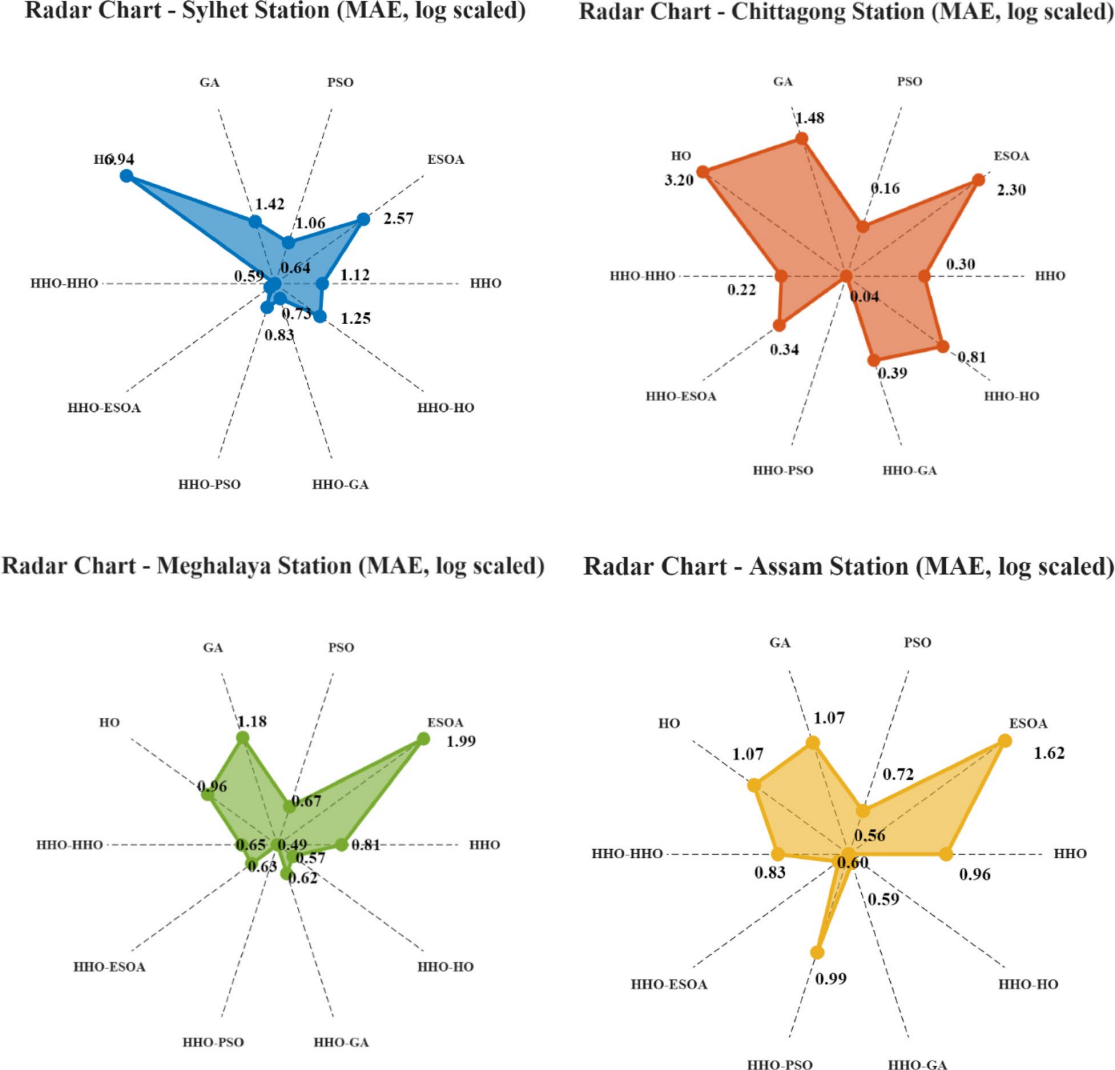
Radar chart based on log-scaled MAE values for comparative analysis of results.

At Sylhet, the performance of different optimization methods varies significantly, as shown in Table 3. For better readability, an MAE-based radar chart, as shown in Fig. 10, is also presented, where axes distinguish single and double phase methods. In the figure, points near the center represent lower prediction errors. Among the independent models, Harris Hawks Optimization (HHO) proves to be the most accurate with an MSE of 2.96, RMSE of 1.72, MAE of 1.12, and an impressive R² value of 0.99. This means HHO is remarkably precise in prediction with almost no error. Egret Swarm Optimization (ESOA), nonetheless, has higher error values (MSE = 25.94, RMSE = 5.09, and R² = 0.91), showing its predictive accuracy is poorer than HHO. Particle Swarm Optimization (PSO) is excellent with an MSE of 4.97, RMSE of 2.23, MAE of 1.06, and R² of 0.98, and it is a highly competitive algorithm though not as accurate as HHO. Genetic Algorithm (GA) ranks second with an MSE of 7.86, RMSE of 2.8, MAE of 1.42, and R² of 0.97, indicating moderate accuracy but lagging behind HHO and PSO. In contrast, Hippopotamus Optimization (HO) is the worst in predictive potential with an MSE of 199.65, RMSE of 14.12, and a much lower R² of 0.34, and hence not well suited for modeling accurately here.

When hybrid models come into play, significant improvements can be seen. The HHO-ESOA model does the best in all aspects, with an MSE of 1.71, RMSE of 1.31, MAE of 0.64, and R² of 0.99, doing better in accuracy compared to any of the individual models. In a similar vein, HHO-HHO does very well as a prediction model, with an MSE of 1.75, RMSE of 1.32, MAE of 0.59, and R² of 0.99, making it another highly effective hybrid model. Other hybrid models, such as HHO-PSO (MSE = 4.92, RMSE = 2.21, R² = 0.98) and HHO-GA (MSE = 1.94, RMSE = 1.39, R² = 0.99), offer competitive accuracy, demonstrating that the integration of HHO with other optimization techniques can further enhance prediction performance. Conversely, HHO-HO (MSE = 5.06, RMSE = 2.24, R² = 0.98) shows a remarkable improvement over HO in isolation but is weaker than the other hybrid models.

#### Results obtained for the Chittagong region

For the Chittagong region, the prediction ability of different optimization techniques is quite different. Among the individual models, Harris Hawks Optimization (HHO) is outstanding, with an MSE of 0.12, an RMSE of 0.34, and an R² value of 0.99, indicating nearly perfect predictions with very little error. Particle Swarm Optimization (PSO) does better, in fact, with an MSE of 0.078, RMSE of 0.27, MAE of 0.16, and an R² of 0.99, the optimal individual model. Genetic Algorithm (GA), while maintaining satisfactory prediction capability, produces higher error measures (MSE = 2.7, RMSE = 1.64, R² = 0.98) compared to HHO and PSO. Egret Swarm Optimization (ESOA) does a lot worse, with an MSE of 6.29, an RMSE of 2.5, and an R² of 0.96, which is less appropriate for precise forecasting in this case. Hippopotamus Optimization (HO) has the worst performance, with a very high MSE of 43.12, RMSE of 6.56, and an R² of 0.78, with weak predictive power and unsuitability for this purpose.

**Table 3:** Results of single and double step Framework.

Station	Algorithm type	Optimizer	MSE	RMSE	MAE	*R* ^2^	Activation function	Neuron
Sylhet		HHO	2.96	1.72	1.12	0.99	relu	3
	Conventional model (Single step)	ESOA	25.94	5.09	2.57	0.91	relu	14
		PSO	4.97	2.23	1.06	0.98	relu	12
		GA	7.86	2.8	1.42	0.97	leakyrelu	10
		HO	199.65	14.12	6.94	0.34	leakyrelu	10
		HHO-HHO	1.75	1.32	0.59	0.99	leakyrelu	4
	Proposed model (Double step)	HHO-ESOA	1.71	1.31	0.64	0.99	leakyrelu	7
		HHO-PSO	4.92	2.21	0.83	0.98	leakyrelu	6
		HHO-GA	1.94	1.39	0.73	0.99	relu	3
		HHO-HO	5.06	2.24	1.25	0.98	relu	10
Chittagong		HHO	0.12	0.34	0.3	0.99	relu	24
	Conventional model (Single step)	ESOA	6.29	2.5	2.3	0.96	leakyrelu	30
		PSO	0.078	0.27	0.16	0.99	relu	24
		GA	2.7	1.64	1.48	0.98	relu	30
		HO	43.12	6.56	3.2	0.78	leakyrelu	10
		HHO-HHO	0.11	0.33	0.22	0.99	relu	15
	Proposed model (Double step)	HHO-ESOA	0.18	0.42	0.34	0.99	leakyrelu	9
		HHO-PSO	0.0083	0.091	0.04	1	leakyrelu	22
		HHO-GA	0.5	0.71	0.39	0.99	leakyrelu	15
		HHO-HO	1.43	1.19	0.81	0.99	relu	23
Meghalaya		HHO	3.17	1.78	0.81	0.98	leakyrelu	1
	Conventional model (Single step)	ESOA	14.92	3.86	1.99	0.93	relu	16
		PSO	2.59	1.61	0.67	0.98	leakyrelu	17
		GA	4.33	2.08	1.18	0.97	‘leakyrelu’	1
		HO	3.68	1.92	0.96	0.98	relu	1
		HHO-HHO	2.95	1.71	0.65	0.98	leakyrelu	1
	Proposed model (Double step)	HHO-ESOA	1.55	1.24	0.63	0.99	relu	1
		HHO-PSO	1.49	1.22	0.49	0.99	leakyrelu	1
		HHO-GA	1.51	1.23	0.62	0.99	relu	1
		HHO-HO	1.43	1.19	0.57	0.99	relu	1
Assam		HHO	2.64	1.62	0.96	0.97	relu	1
	Conventional model (Single step)	ESOA	4.68	2.16	1.62	0.95	‘leakyrelu’	11
		PSO	2.48	1.57	0.72	0.97	‘leakyrelu’	20
		GA	4.48	2.11	1.07	0.95	‘leakyrelu’	1
		HO	2.6	1.61	1.07	0.97	‘leakyrelu’	1
		HHO-HHO	3.2	1.78	0.83	0.96	‘leakyrelu’	1
	Proposed model (Double step)	HHO-ESOA	1.12	1.06	0.6	0.98	‘leakyrelu’	1
		HHO-PSO	2.06	1.43	0.99	0.97	‘leakyrelu’	1
		HHO-GA	1.59	1.26	0.59	0.98	‘leakyrelu’	1
		HHO-HO	1.58	1.26	0.56	0.98	‘leakyrelu’	1

For hybrid models, improvements are observed to be high. The HHO-HHO model achieves a low MSE of 0.11, RMSE of 0.33, and R² of 0.99, vindicating its strength. The HHO-PSO hybrid model is better than all the models with an MSE of 0.0083, an RMSE of 0.091, and an R² of 1, nearly being perfect in prediction accuracy. The other hybrid models, such as HHO-ESOA (MSE = 0.18, RMSE = 0.42, R² = 0.99) and HHO-GA (MSE = 0.5, RMSE = 0.71, R² = 0.99), are also very good at making predictions but slightly less so than HHO-PSO. The HHO-HO hybrid (MSE = 1.43, RMSE = 1.19, R² = 0.99) is better than HO on its own but worse than the other hybrid combinations.

#### Results obtained for the Meghalaya region

For Meghalaya, predictive accuracy with different optimization techniques varies in individual and hybrid models. Among the individual approaches, Harris Hawks Optimization (HHO) performs well in terms of predictive performance with an MSE of 3.17, RMSE of 1.78, and R² of 0.98, which reflects high predictive accuracy. Particle Swarm Optimization (PSO) provides nearly similar values, with an MSE of 2.59, RMSE of 1.61, and R² of 0.98, making it a serious contender for HHO. Genetic Algorithm (GA) produces larger error values than HHO and PSO, with an MSE of 4.33, RMSE of 2.08, and R² of 0.97, reflecting a slight decline in precision. Egret Swarm Optimization (ESOA), however, shows a substantial decrease in performance with an MSE of 14.92, RMSE of 3.86, and an R² of 0.93 and hence is less effective for this particular task. Hippopotamus Optimization (HO) has the same performance as HHO but is ever so slightly less effective with an MSE of 3.68, RMSE of 1.92, and R² of 0.98.

When hybrid models are considered, dramatic accuracy improvements are observable. HHO-HHO, MSE = 2.95, RMSE = 1.71, R² = 0.98, possesses a strong predictive power. Hybrid blends with other algorithms work even better. HHO-ESOA (MSE = 1.55, RMSE = 1.24, R² = 0.99) and HHO-PSO (MSE = 1.49, RMSE = 1.22, R² = 0.99) are found to possess much higher accuracy, hence further cementing their feasibility. The other hybrid variants, HHO-GA (MSE = 1.51, RMSE = 1.23, R² = 0.99) and HHO-.

HO (MSE = 1.43, RMSE = 1.19, R² = 0.99), are also shown to have decent prediction capabilities with HHO-PSO emerging as one of the finest hybrid techniques.

#### Results obtained for the Assam region

In Assam, the performance of the predictive capability of different optimization algorithms varies in success. Out of the single models, Harris Hawks Optimization (HHO) is successful with an MSE of 2.64, RMSE of 1.62, MAE of 0.96, and R² of 0.97, indicating valid predictive potential. Particle Swarm Optimization (PSO) also exhibits a comparable level of performance, with an MSE of 2.48, RMSE of 1.57, and R² of 0.97, which makes PSO a good alternative to HHO. On the other hand, the Genetic Algorithm (GA) decreases its performance with an MSE of 4.48, RMSE of 2.11, and R² of 0.95, reflecting a moderate increase in error. Furthermore, Egret Swarm Optimization (ESOA), with an MSE of 4.68, RMSE of 2.16, and R² of 0.95, is equal to that of GA but less effective than HHO and PSO. Hippopotamus Optimization (HO), with MSE of 2.6, RMSE of 1.61, and R² of 0.97, is revealed to be as effective and even better in performance than HHO.

In the consideration of hybrid models, predictive accuracy improves significantly. HHO-ESOA, with an MSE of 1.12, RMSE of 1.06, and R² of 0.98, is found to be the top-performing combination, minimizing error values substantially and improving precision. HHO-HHO (MSE = 3.2, RMSE = 1.78, R² = 0.96) and HHO-PSO (MSE = 2.06, RMSE = 1.43, R² = 0.97) are also among the top-performing combinations, with the latter exhibiting a considerable improvement over individual model. The remaining hybrid models, such as HHO-GA (MSE = 1.59, RMSE = 1.26, R² = 0.98) and HHO-HO (MSE = 1.58, RMSE = 1.26, R² = 0.98), demonstrate high predictive accuracy, indicating the merits of combining optimization techniques for enhanced forecasting.

#### Station-wise analysis of best-performing optimization models

Out of the models from the Sylhet station, the hybrid HHO-ESOA optimizer was the overall best performing, which yielded an MSE of 1.71, MAE of 0.64, RMSE of 1.31, and R² value of 0.99. Notably, this model was also able to correctly forecast the peak rainfall values, reflecting its effectiveness in handling high fluctuation situations. Table 3 presents the best-performing models for each station. The HHO-PSO optimizer gave outstanding results at the Chittagong station as well, with an exceptionally low MAE of 0.04, MSE of 0.0083, and RMSE of 0.091, along with a very close-to-perfect R² value of 1.00, meaning very accurate predictions across the whole range, including peak points. At Meghalaya, the rainiest of all stations, the HHO-HO optimizer performed flawlessly, recording the optimum MSE of 1.43, MAE of 0.57, and RMSE of 1.19, while having a very high R² value of 0.99. The model was particularly effective in modeling sharp increases in rainfall, detecting peak events with reasonable accuracy. Similarly, at the Assam station, hybrid optimizers outperformed others, and the HHO-ESOA model gave the best performance: MSE of 1.12, RMSE of 1.06, MAE of 0.60, and R² of 0.98. It also demonstrated strong capability in detecting the maximum rainfall intensities, thereby improving the prediction accuracy during the critical periods.

#### Result comparison between single step and dual step optimization framework

The Table 4 provides comparative information about various optimization techniques applied for predictive accuracy improvement at four stations: Sylhet, Chittagong, Meghalaya, and Assam. All optimizers (HHO, HO, ESOA, GA, PSO) are compared with a two-step hybrid approach (HHO-HHO, HHO-HO, HHO-ESOA, HHO-GA, HHO-PSO). Negative percentage values indicate improvement, i.e., the double-step optimization method reduces the error of the single optimizer. Outputs indicate that hybrid models outperform single optimizers. Figure 10 presents a radar chart showcasing the performance enhancements obtained through the use of two-step optimized models.

**Table 4:** Error reduction through hybrid optimization systems.

Station	Optimizer	MSE change (%)	RMSE change (%)	MAE change (%)
Sylhet	HHO vs. HHO-HHO	−40.54%	−23.26%	−47.32%
	ESOA vs. HHO-ESOA	−93.39%	−23.13%	−42.86%
	HO vs. HHO-HO	−97.46%	−84.22%	−81.99%
	GA vs. HHO-GA	−75.34%	−50.71%	−48.60%
	PSO vs. HHO-PSO	−1.00%	−0.90%	−21.70%
Chittagong	HHO vs. HHO-HHO	−8.33%	−3.33%	−26.67%
	ESOA vs. HHO-ESOA	−97.10%	−85.20%	−85.83%
	HO vs. HHO-HO	−80.11%	−63.40%	−55.69%
	GA vs. HHO-GA	−81.48%	−57.48%	−63.83%
	PSO vs. HHO-PSO	−89.33%	−66.67%	−75.00%
Meghalaya	HHO vs. HHO-HHO	−6.96%	−4.14%	−19.75%
	ESOA vs. HHO-ESOA	−89.57%	−67.01%	−68.52%
	HO vs. HHO-HO	−61.48%	−38.90%	−48.25%
	GA vs. HHO-GA	−50.38%	−40.38%	−47.46%
	PSO vs. HHO-PSO	−42.47%	−24.22%	−29.63%
Assam	HHO vs. HHO-HHO	−17.93%	−10.92%	−13.54%
	ESOA vs. HHO-ESOA	−76.09%	−48.84%	−62.27%
	HO vs. HHO-HO	−65.73%	−60.25%	−47.79%
	GA vs. HHO-GA	−64.61%	−44.51%	−65.47%
	PSO vs. HHO-PSO	−17.74%	−8.89%	−30.56%

For Sylhet, HHO-HHO reduces MSE by 40.54%, RMSE by 23.26%, and MAE by 47.32%, which indicates that using HHO twice enhances performance. The hybrid approach HHO-ESOA greatly improves ESOA, with a 93.39% reduction in MSE, demonstrating that hybridization greatly improves ESOA. Surprisingly, HHO-HO gains the highest performance improvement in Sylhet, reducing MSE, RMSE, and MAE by 97.46%, 84.22%, and 81.99%, respectively, demonstrating that the synergy between the global exploration of HHO and the local tuning of HO is highly effective. Similarly, HHO-GA and HHO-PSO improve results, but the PSO-based hybrid only marginally improves in this station. In Chittagong, the hybrid models are good, particularly HHO-ESOA, which reduces MSE, RMSE, and MAE by 97.10%, 85.20%, and 85.83%, respectively. HHO-HO and HHO-GA also show considerable improvements, reducing MSE by 80.11% and 81.48%, respectively, as proof that GA is further improved. Unexpectedly, HHO-PSO witnesses a massive 89.33% reduction in MSE, which is in contrast to Sylhet, confirming that PSO’s performance enhancement is dataset-oriented. Meghalaya also observes the same trend but with considerably smaller gains. HHO-ESOA again records the most MSE reduction at 89.57%, followed by other combinations such as HHO-HO and HHO-GA that have a moderate boost. In the case of Assam, two-step approaches maintain their advantage, with HHO-ESOA reducing MSE by 76.09%, HHO-HO by 65.73%, and HHO-GA by 64.61%.

**Fig. 11:**
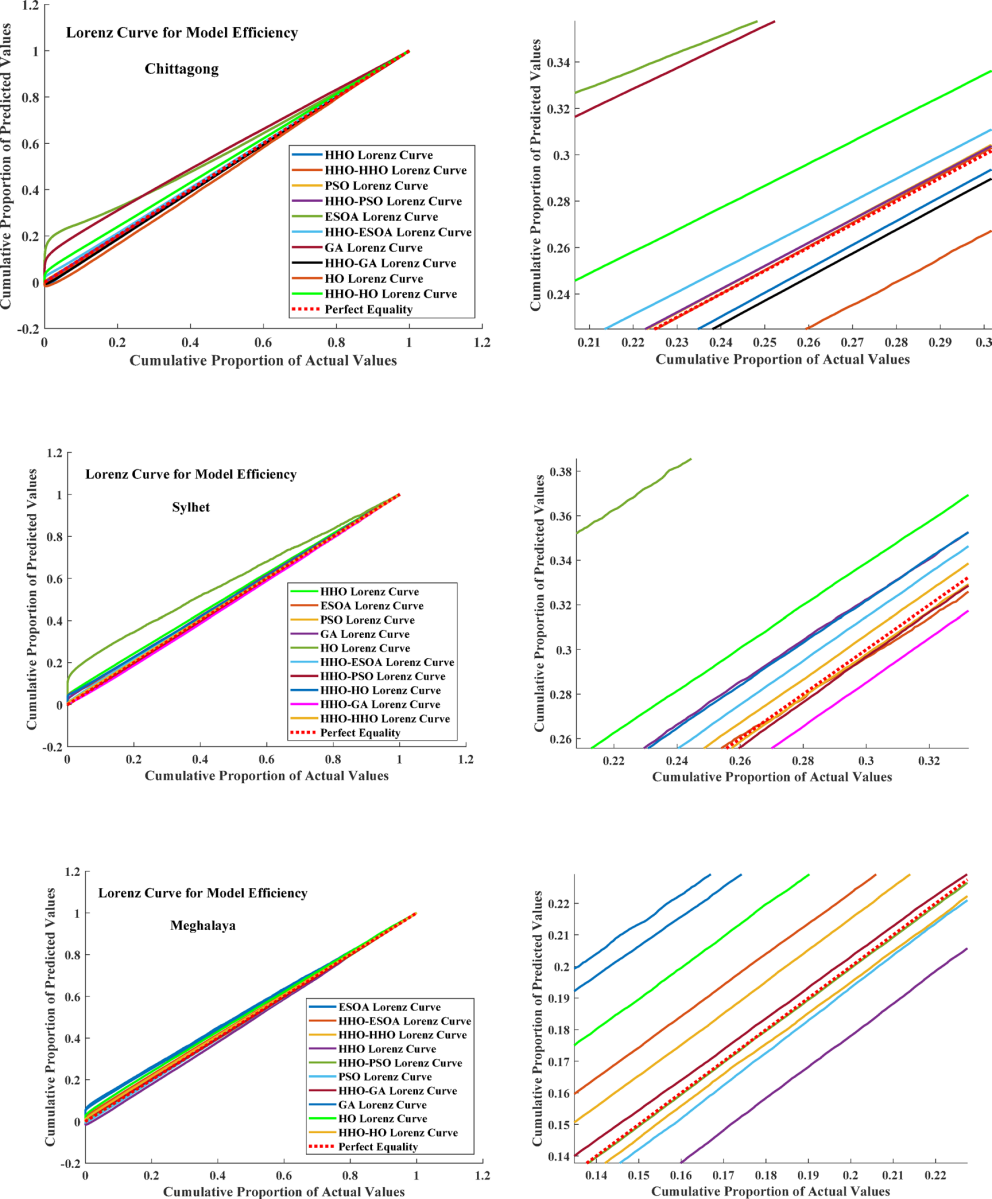

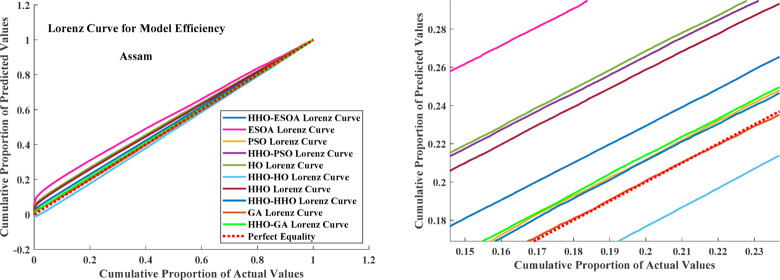
Lorenz curve for actual and predicted values.

The result confirms that employing a double-step optimization approach enhances prediction accuracy significantly by reducing errors in all key metrics. The result of all double-step and single-step models is depicted in Fig. 11 with the Lorenz Curve. The Lorenz Curve is often used to compare model efficiency by graphing the cumulative proportion of actuals vs. the cumulative proportion of predictions. The Perfect Equality Line is an ideal 1:1 relation, and deviance from it detects variation in model performance.

### Justification for employing a dual-step optimization approach

Two-step optimization outperforms one-step optimization in Artificial Neural Network (ANN) weight optimization for rainfall modeling because of several critical benefits of search efficiency, convergence rate, and local minimum robustness. The primary reasons why hybrid two-step approaches (e.g., HHO-HHO, HHO-HO, HHO-ESOA, HHO-GA, HHO-PSO) perform better than single optimizers (HHO, HO, ESOA, GA, PSO) in rainfall prediction models are: Single-step optimization algorithms tend to fall short in striking a balance between exploration (global search for improvement) and exploitation (local tuning for precision). Double-step optimization, however, leverages the best of two optimizers, where the first step involves global search and the second involves fine-tuning the weights locally. For instance, in HHO-HO, HHO efficiently explores the solution space, and HO fine-tunes the outcomes so that premature convergence is avoided. This leads to more accurate rainfall predictions.

Rainfall data is extremely nonlinear, and ANN training is typically plagued by local minima traps, and hence poor performance. One optimizer sometimes cannot escape from such local minima, and the predictive accuracy is limited. A two-stage hybrid optimizer such as HHO-ESOA employs HHO initially to roughly search for the best ANN weights and then ESOA to refine to avoid the network from becoming stuck in a local minimum. Optimizers may also converge more slowly, i.e., require more iterations to get to the optimal ANN weights. A two-stage technique accelerates convergence by first approximating an optimal region and then refining it in detail. For instance, HHO-GA applies the good exploratory capabilities of HHO first and then GA for more precise weight selection. It reduces training time while improving prediction stability. On the other hand, Single-step optimizers overfit to training data, especially when they are optimizing ANN weights for highly fluctuating rainfall data. Hybrid optimizations help prevent overfitting by making the first phase find a stable global solution and the second phase enhances the network’s generalization ability. This makes the model less sensitive to noise in real rainfall predictions.

### Sensitivity analysis on the number of hidden neurons

One of the most significant parameters influencing Artificial Neural Network (ANN) performance is the number of hidden neurons, as noted in previous research^90^. To investigate this influence, a sensitivity analysis was conducted by expanding the optimization range of hidden neurons from 1 to 30 to 1–100. The objective was to assess whether model capacity increases would lead to improved prediction performance at different stations.

The results were varied depending on the optimizer and station. Significant improvement was observed for HO (98.25% MSE reduction), GA (58.52%), and ESOA (57.67%) at increased ranges of neurons. There were, however, some models such as HHO (− 55.23%), HHO-HHO (− 105.74%), and HHO-GA (− 63.74%) with increased errors indicating a performance drop at increased neuron sizes. For the Chittagong station, HHO-GA (98.08% error reduction), HO (96.67%), and GA (93.86%) had higher neuron search space. Conversely, some of them—particularly HHO-PSO (− 698.80%), HHO-ESOA (− 523.67%), and PSO (− 2,947.56%)—experienced sudden spikes in error, which means their best settings were already at the lower neuron scope.

In Meghalaya, expanding the number of neurons improved models such as GA (60.25%), ESOA (39.82%), and HHO-HHO (45.93%), whereas models such as HHO-ESOA (− 119.42%) and HHO-HO (− 113.14%) saw error growth. Similarly, at the Assam station, HHO-HHO (52.02%), PSO (32.43%), and HHO-PSO (27.15%) were improved with a rise in neurons, whereas HHO (− 170.08%) and HHO-GA (− 32.70%) saw error growth.

**Fig. 12:**
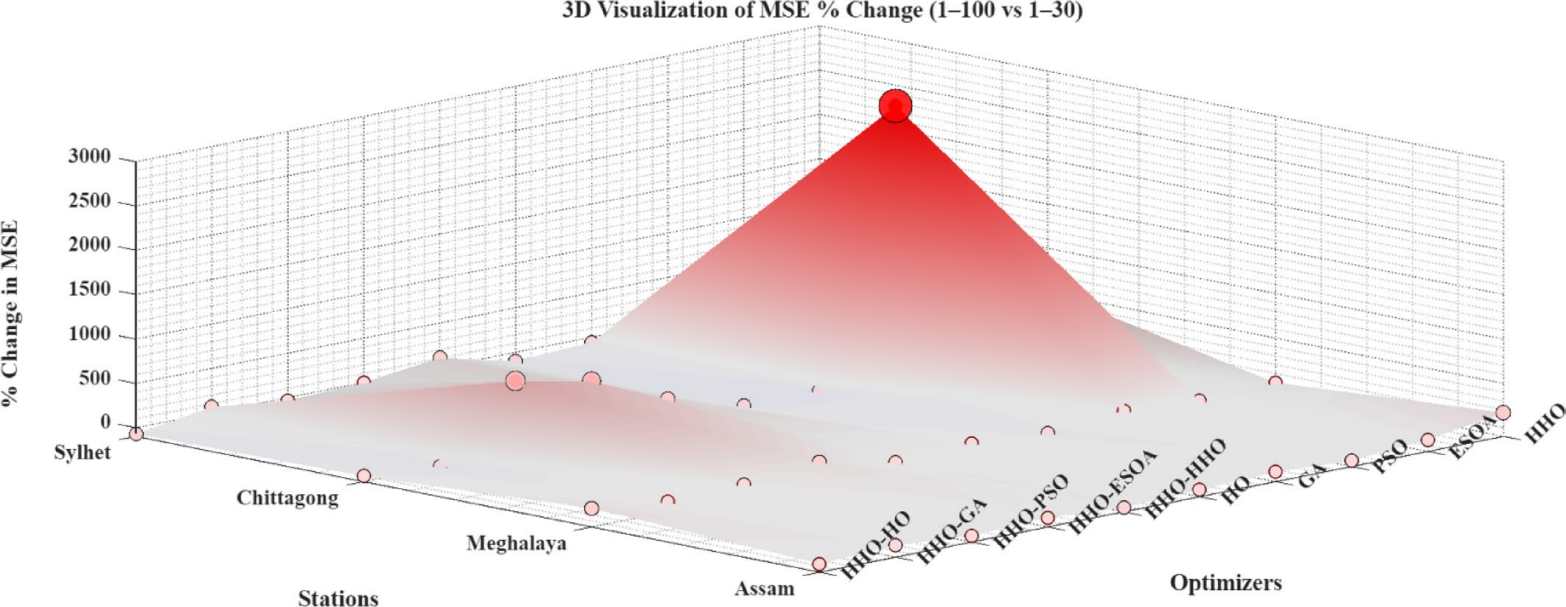
3D visualization of MSE (%) change.

**Fig. 13:**
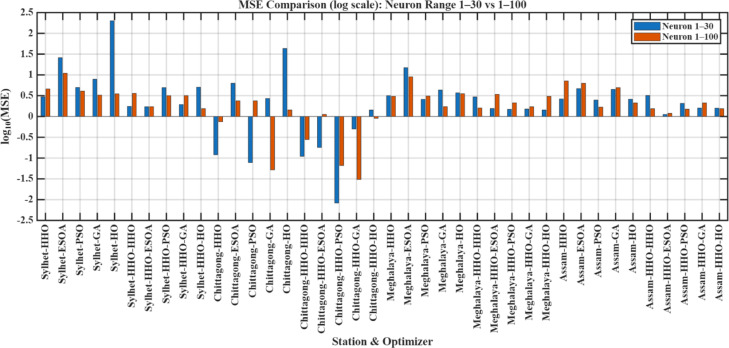
Log-scale MSE comparison for neuron ranges 1–30 vs. 1–100.

**Fig. 14:**
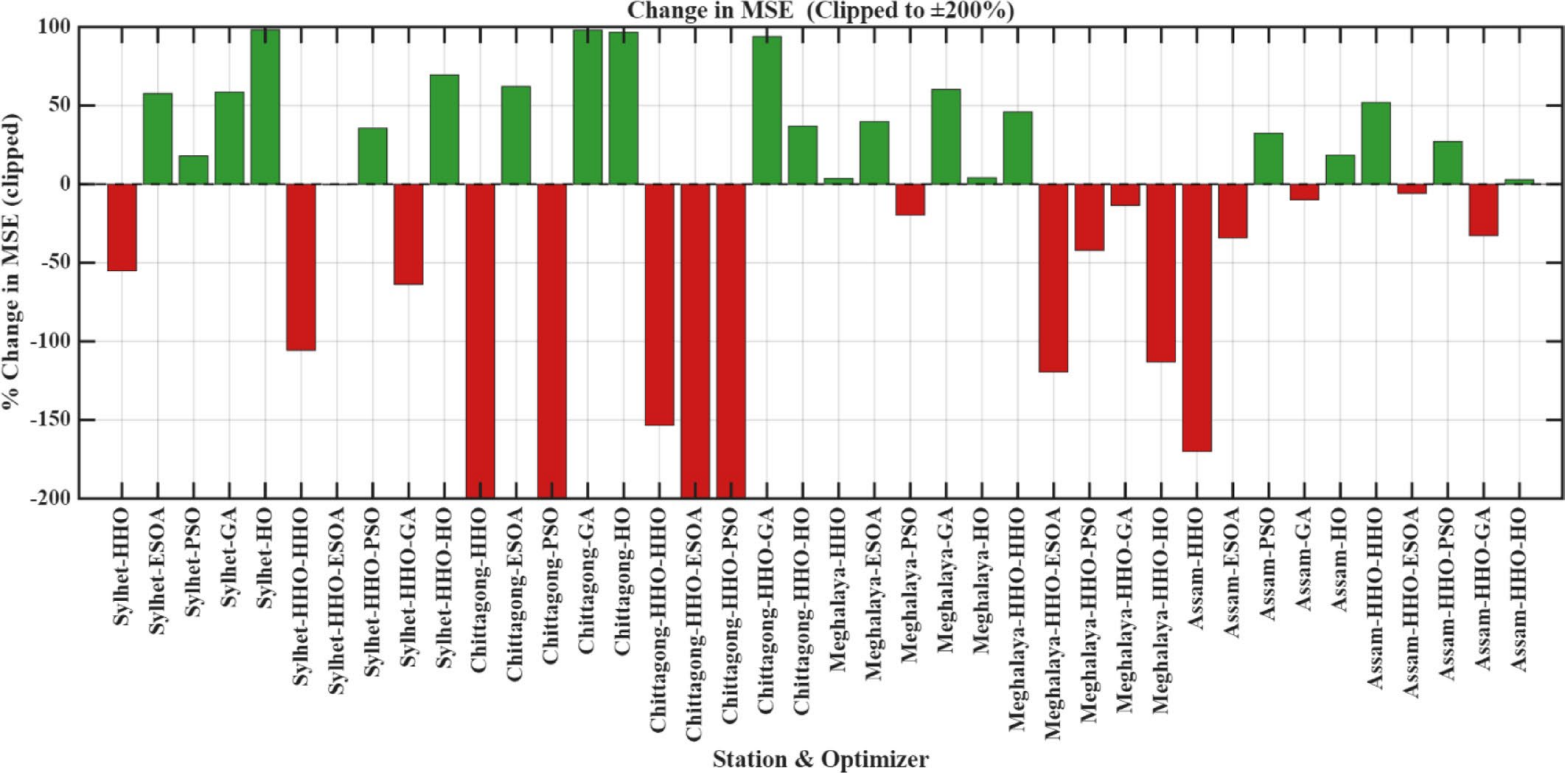
Percentage change in MSE with increased neurons (clipped at ± 200% for readability).

The results indicate that the influence of hidden neuron number is highly sensitive to both optimizer choice and the character of available data. Figure 12 (3D) illustrates the influence of varying the number of hidden neurons on the performance of the model, indicating the role of network complexity in prediction accuracy. While additional neurons may be utilized to enhance performance in certain models, it may also result in overfitting or instability in other models. Therefore, careful tuning of the quantity of hidden neurons is required to balance model accuracy and generalizability. Figure 13 presents log-scaled MSE values for all configurations, where positive axis bars indicate values > 1 and negative bars indicate MSEs < 1 because of the logarithmic scale. Figure 14 displays the percent change in MSE with more neurons, cut off at ± 200% for legibility. Green bars represent improvements (error decrease), and red bars represent degradation in performance. From these results, it is clear that the effect of increasing the number of hidden neurons is vastly varied depending on both the optimizer used and the station in question. A positive percentage means performance improvement, and a negative value represents an increase in the prediction error.

### Limitations and future recommendation

The proposed methodology performs well in the development of a rainfall prediction model for four geological stations: Chittagong, Sylhet, Assam, and Meghalaya. Although the framework presents promising results, some limitations are found, which are explained below:

- Resolution of the data in google earth engine (GEE): Google Earth Engine (GEE) was used in this research to retrieve rainfall data. The resolution of the data offered by GEE is comparatively low. For the improvement of real-time rainfall modeling reliability, more sophisticated platforms offering higher-resolution datasets should be used in future studies.
- Improving rainfall prediction with more variables: This work accounts for rainfall data only for modeling purposes. In addition to improving the performance and generalizability of the proposed rainfall prediction model, the incorporation of other meteorological data such as temperature, relative humidity, and wind speed is recommended. Such parameters have been found to influence atmospheric moisture content and precipitation characteristics, and their addition is likely to provide a better representation of the underlying physical mechanisms. By enriching the input feature space, the ability of the model to capture complex interactions and temporal relationships can be significantly enhanced, ultimately leading to more accurate and reliable rainfall forecasts.
- Refining the ideal number of components in non-negative matrix factorization (NMF): As part of data preprocessing, training of NMF models was performed. The selection of ideal components in NMF is critical for model accuracy. The study employed the use of the reconstruction error plot and its derivative forms to refine the process of selecting the ideal components, up to 100 components. Future studies are encouraged to explore alternative approaches to determining the optimal number of components that can further enhance the reliability of real-time rainfall forecasting.
- Artificial neural network (ANN) parameter optimization: In the optimization procedure, the range of transfer functions and neurons for the ANN model was set to a small value. To provide greater flexibility in the model, it is recommended to enhance the range of hidden layers and transfer functions for future research. Moreover, the implementation of recently proposed metaheuristic optimization algorithms can be employed to enhance model performance for real-time forecasting.
- Population size in optimization algorithms: Throughout this study, different optimization algorithms like Harris Hawks Optimization (HHO), Hippopotamus Optimization (HO), Egret Swarm Optimization Algorithm (ESOA), Genetic Algorithm (GA), and Particle Swarm Optimization (PSO) and their hybrid versions were applied. However, optimization was executed with a small population size. Augmenting the initial solution space could lead to more optimum weight values for the ANN model.
- Dual-Step optimization strategy: The study employed a dual-step optimization strategy with HHO as the first-stage optimizer. For further enhancement of optimization performance, future research must explore different configurations with other algorithms as the first-stage optimizer.
- Employing deep learning model: Even though this study employs a standard ANN model, it acknowledges recent developments in advanced deep learning models such as LSTM, RNN, and CNN that have demonstrated great success in time series forecasting. Future research needs to include these models as benchmarks, especially in handling complex rainfall dynamics. Overcoming this gap would help de-mystify the relative strengths of hybrid metaheuristic-tuned ANN and advanced deep learning models in rainfall forecasting.

By addressing these limitations, future research can render the proposed methodology more robust and versatile for real-time rainfall forecasting applications.

## Conclusion

This study addresses the challenge of noisy and complex rainfall patterns by using advanced signal processing and recently introduced metaheuristic optimization algorithms to enhance the optimization of Artificial Neural Network (ANN) weights. The proposed two-stage optimization method integrates Non-negative Matrix Factorization (NMF) with well-known (GA, PSO) and recently developed metaheuristic algorithms, such as Harris Hawks Optimization (HHO), Hippopotamus Optimization (HO), and Egret Swarm Optimization Algorithm (ESOA). The results demonstrate the potentiality of the dual-step hybrid model compared to traditional single-step optimization approaches, improving model accuracy significantly. Results exhibit considerable improvement in reduction Mean Squared Error (MSE), ranging from 1.00% to 97.46% in Sylhet, 8.33% to 97.10% in Chittagong, 6.96% to 89.57% in Meghalaya, and 17.74% to 76.09% in Assam over single-step optimized models. To provide transparency and facilitate further improvement in this area, this study presents step-by-step pseudocode, allowing future researchers and practitioners to easily replicate, verify, or expand the suggested methodology. These findings prove the robustness of the presented method in the case of extreme rainfall forecasting situations. Also, the sensitivity analysis of the hidden neurons proves that the impact of neuron count is sensitive to the optimizer and type of the dataset. While the work realizes promising outcomes, some limitations have been identified; these are the resolution limitation of GEE rainfall data, a more fine-grained selection of NMF components, partial tuning of ANN parameters, small sizes in metaheuristic algorithms, and insufficient full exploration of hybrid combinations. All these should be remedied in future work using higher-resolution datasets, adaptive NMF methods, more extensive ANN architecture search, and more heterogeneous hybridization options. It is also recommended to use advanced deep learning models such as LSTM, RNN, and CNN, which have shown great promise in time series prediction. Future studies would need to use these models as a benchmark, particularly for understanding the complexities of rainfall dynamics. Overall, the proposed methodology shows strong promise for real-time rainfall forecasting and has practical implications for improving flood management policies in Bangladesh.

## Supplementary Information

Below is the link to the electronic supplementary material.

Supplementary Material 1

## Data Availability

The datasets used in this study were obtained from the Google Earth Engine platform (https://earthengine.google.com/), which provides public access to satellite and environmental datasets. The processed datasets generated and analyzed during the current study are available from the corresponding author, Shuvendu Pal Shuvo (shuvenduce@gmail.com), upon reasonable request.

## References

[1] Mirza, M. M. Q. Three recent extreme floods in Bangladesh: A hydro-meteorological analysis. *Nat. Hazards* **28**(1), 35–64. 10.1023/A:1021169731325 (2003).

[2] Dewan, A. M., Nishigaki, M. & Komatsu, M. *Floods in Bangladesh A Comparative Hydrological Investigation on Two Catastrophic Events.* (2003).

[3] Kabir, M. H. & Hossen, M. N. *Impacts of flood and its possible solution in Bangladesh.* https://www.researchgate.net/publication/336146425 (2019).

[4] https://www.dhakatribune.com/bangladesh/nation/352004/why-does-the-sylhet-region-see-repeated-flooding.

[5] https://www.thedailystar.net/news/bangladesh/news/three-lakh-stranded-flash-flood-hits-4-upazilas-sylhet-3622811.

[6] Al Mahbub, H., Muhibbullah, M. & Sarwar, I. U*rban Flooding and Water Pollution: A Geographical Study on Port City Chattogram, Bangladesh.* https://www.researchgate.net/publication/384501648.

[7] Bari, S. H., Rahman, M. T., Hussain, M. M. & Ray, S. Forecasting monthly precipitation in Sylhet City using ARIMA model. **7** 1 www.iiste.org. (2015).

[8] Pandey, P. K., Pandey, H., Tripura, & Pandey, V. Improving prediction accuracy of rainfall time series by hybrid SARIMA–GARCH modeling. *Nat. Resour. Res.* **28**(3), 1125–1138. 10.1007/s11053-018-9442-z (2019).

[9] Unnikrishnan, P. & Jothiprakash, V. Hybrid SSA-ARIMA-ANN Model for Forecasting Daily Rainfall. *Water Resources Management* **34**(11), 3609–3623. 10.1007/s11269-020-02638-w (2020).

[10] Abd-Elhamid, H. F. et al. Rainfall forecasting in arid regions in response to climate change using ARIMA and remote sensing. *Geomatics, Natural Hazards and Risk*10.1080/19475705.2024.2347414 (2024).

[11] Ray, S., Das, S. S., Mishra, P. & Al Khatib, A. M. G. ime Series SARIMA Modelling and Forecasting of Monthly Rainfall and Temperature in the South Asian Countries. *Earth Syst. Environ.* **5**(3), 531–546 (2021).

[12] Barman, U., Das, D., Dutta, M., Das, K. M. & Chowdhury, S. Enhancing Monsoon Rainfall Forecasting in Assam and Meghalaya: A Time Series Analysis. https://www.researchgate.net/publication/382060787.

[13] Mahmud, I., Bari, S. H. & Ur Rahman, M. T. Monthly rainfall forecast of Bangladesh using autoregressive integrated moving average method. *Environ. Eng. Res.* **22** (2), 162–168. 10.4491/eer.2016.075 (2017).

[14] Masum, M. H., Islam, R., Hossen, M. A. & Akhie, A. A. Time Series Prediction of Rainfall and Temperature Trend using ARIMA Model. *J. Sci. Res.* **14**(1), 215–227. 10.3329/jsr.v14i1.54973 (2022).

[15] Moharana, L., Sahoo, A. & Ghose, D. K. Prediction of Rainfall Using Hybrid SVM-HHO Model, in *IOP Conference Series: Earth and Environmental Science*, Institute of Physics, https://doi.org/10.1088/1755-1315/1084/1/012054 (2022).

[16] Ahmed Osmani, S. et al. *Prediction of Rainfall using Machine Learning Algorithms for Different Districts of Meghalaya.* https://www.researchgate.net/publication/349707159.

[17] Banik, S., Chanchary, F. H., Khan, K., Rouf, A. & Anwer, M. N*eural Network and Genetic Algorithm Approaches for Forecasting Bangladeshi Monsoon Rainfall.* http://www.barc.gov.bd/database.

[18] Goyal, M. K. Monthly rainfall prediction using wavelet regression and neural network: an analysis of 1901–2002 data, Assam, India. *Theor. Appl. Climatol.* **118**(1–2), 25–34. 10.1007/s00704-013-1029-3 (2014).

[19] Biplob, M. B. & Haque, M. M. An Efficient Machine Learning Classification Model for Rainfall Prediction in Bangladesh,. In *Lecture Notes in Networks and Systems* (Springer Science and Business Media Deutschland GmbH, 2024). 10.1007/978-981-99-8937-9_12.

[20] Rachidi, S., El Mazoudi, E. H., El Alami, J., Jadoud, M. & Er-Raki, S. Assessment and Comparison of Satellite-Based Rainfall Products: Validation by Hydrological Modeling Using ANN in a Semi-Arid Zone. *Water (Switzerland)*10.3390/w15111997 (2023).

[21] Lee, J., Kim, C. G., Lee, J. E., Kim, N. W. & Kim, H. Application of artificial neural networks to rainfall forecasting in the Geum River Basin, Korea. *Water (Switzerland)*10.3390/w10101448 (2018).

[22] Usman Saeed Khan, M., Mohammad Saifullah, K., Hussain, A. & Mohammad Azamathulla, H. Comparative analysis of different rainfall prediction models: A case study of Aligarh City, India. *Results Eng.*10.1016/j.rineng.2024.102093 (2024).

[23] Hamidi, O. et al. A comparative study of support vector machines and artificial neural networks for predicting precipitation in Iran. *Theor. Appl. Climatol* **119**, 3–4. 10.1007/s00704-014-1141-z (2015).

[24] Ghamariadyan, M. & Imteaz, M. A. A wavelet artificial neural network method for medium-term rainfall prediction in Queensland (Australia) and the comparisons with conventional methods. *Int. J. Climatol.* **41**, E1396–E1416. 10.1002/joc.6775 (2021).

[25] Mishra, N., Soni, H. K., Sharma, S. & Upadhyay, A. K. Development and analysis of Artificial Neural Network models for rainfall prediction by using time-series data. *Int. J. Intell. Syst. Appl.* **10**(1), 16–23. 10.5815/ijisa.2018.01.03 (2018).

[26] Kandasamy, O. & Kannan, M. N. S. E. R. R. B. Rainfall prediction using artificial neural networks and machine learning algorithms over the Coimbatore region. *J. Water Climate Change*10.2166/wcc.2025.576 (2025).

[27] Hudnurkar, S. & Rayavarapu, N. On the performance analysis of rainfall prediction using mutual information with artificial neural network. *Int. J. Electr. Comput. Eng.* **13**(2), 2101–2113. 10.11591/ijece.v13i2.pp2101-2113 (2023).

[28] *Proceedings of the International Conference on Electronics and Sustainable Communication Systems (ICESC 2020): 02–04, July 2020*. [IEEE], (2020).

[29] Dash, Y., Mishra, S. K. & Panigrahi, B. K. Rainfall prediction for the Kerala state of India using artificial intelligence approaches. *Comput. Electr. Eng.* **70**, 66–73. 10.1016/j.compeleceng.2018.06.004 (2018).

[30] Darji, M. P., Dabhi, V. K. & Prajapati, H. B. Rainfall forecasting using neural network: A survey, in *Conference Proceeding – 2015 International Conference on Advances in Computer Engineering and Applications, ICACEA 2015*, I*nstitute of Electrical and Electronics Engineers Inc*., 706–713 https://doi.org/10.1109/ICACEA.2015.7164782 (2015).

[31] Pour, S. H., Shahid, S., Chung, E. S. & Wang, X. J. Model output statistics downscaling using support vector machine for the projection of spatial and temporal changes in rainfall of Bangladesh. *Atmos. Res.* **213**, 149–162. 10.1016/j.atmosres.2018.06.006 (2018).

[32] Satriadi, A. & Handoyo, G. Time series analysis and prediction of climate variables of Southern Java waters using support vector regression. *Ecol. Eng. Environ. Technol.* **26** (1), 177–186. 10.12912/27197050/195797 (2025).

[33] Yu, P. S., Yang, T. C., Chen, S. Y., Kuo, C. M. & Tseng, H. W. Comparison of random forests and support vector machine for real-time radar-derived rainfall forecasting. *J. Hydrol. (Amst)*. **552**, 92–104. 10.1016/j.jhydrol.2017.06.020 (2017).

[34] Feng, Q., Wen, X. & Li, J. Wavelet Analysis-Support vector machine coupled models for monthly rainfall forecasting in arid regions. *Water Resour. Manage*. **29** (4), 1049–1065. 10.1007/s11269-014-0860-3 (2015).

[35] Shenify, M. et al. Precipitation Estimation using support vector machine with discrete wavelet transform. *Water Resour. Manage*. **30** (2), 641–652. 10.1007/s11269-015-1182-9 (2015).

[36] Du, J., Liu, Y., Yu, Y. & Yan, W. A prediction of precipitation data based on Support Vector Machine and Particle Swarm Optimization (PSO-SVM) algorithms. *Algorithms*10.3390/a10020057 (2017).

[37] Reddy, P. C. S., Sucharitha, Y. & Narayana, G. S. Development of rainfall forecasting model using machine learning with singular spectrum analysis. *IIUM Eng. J.* **23** (1), 172–186. 10.31436/IIUMEJ.V23I1.1822 (2022).

[38] Kundu, S., Khare, D. & Mondal, A. Future changes in rainfall, temperature and reference evapotranspiration in the central India by least square support vector machine. *Geosci. Front.* **8** (3), 583–596. 10.1016/j.gsf.2016.06.002 (2017).

[39] Hasan, N., Nath, N. C. & Rasel, R. I. *A Support Vector Regression Model for Forecasting Rainfall*.

[40] Rahman, A. U. et al. Rainfall Prediction System Using Machine Learning Fusion for Smart Cities. *Sensors*10.3390/s22093504 (2022). 35591194 10.3390/s22093504 PMC9099780

[41] Barrera-Animas, A. Y. et al. Rainfall prediction: A comparative analysis of modern machine learning algorithms for time-series forecasting. *Mach. Learn. Appl.* **7**, 100204. 10.1016/j.mlwa.2021.100204 (2022).

[42] Ridwan, W. M. et al. Rainfall forecasting model using machine learning methods: case study Terengganu, Malaysia. *Ain Shams Eng. J.* **12** (2), 1651–1663. 10.1016/j.asej.2020.09.011 (2021).

[43] Chhetri, M., Kumar, S., Roy, P. P. & Kim, B. G. Deep BLSTM-GRU model for monthly rainfall prediction: A case study of Simtokha, Bhutan. *Remote Sens. (Basel)*. **12**, 1–13. 10.3390/rs12193174 (2020).

[44] Zhao, W., Zhang, Z., Khodadadi, N. & Wang, L. A deep learning model coupled with metaheuristic optimization for urban rainfall prediction. *J. Hydrol. (Amst)*10.1016/j.jhydrol.2024.132596 (2025).

[45] Roy, S. K. et al. EVNN-GRFN integrated with BFGS-ARMA for rainfall prediction in Bangladesh. *Earth Sci. Inf.*10.1007/s12145-024-01672-1 (2025).

[46] Khosravi, K., Farooque, A. A., Bateni, S. M., Jun, C. & Dhiman, J. Prediction of three vital rainfall characteristics: Advanced hybrid tree- or lazy-based learner?. *Results Eng.*10.1016/j.rineng.2024.103840 (2025).

[47] Küllahcı, K. & Altunkaynak, A. Maximizing daily rainfall prediction accuracy with maximum overlap discrete wavelet transform-based machine learning models. *Int. J. Climatol.* **44** (10), 3405–3426. 10.1002/joc.8530 (2024).

[48] Coutinho, E. R. et al. Multi-Step forecasting of meteorological time series using CNN-LSTM with decomposition methods. *Water Resour. Manage*. 10.1007/s11269-025-04102-z (2025).

[49] Darji, M., Dave, J. A., Oza, A. D., Kumar, S. & Kumar, R. An innovative method for improving rainfall prediction in Gujarat state through a fusion model DWT, 1DCNN and LSTM. *Multidisciplinary Sci. J.*10.31893/multiscience.2025109 (2025).

[50] Chau, K. W. & Wu, C. L. A hybrid model coupled with singular spectrum analysis for daily rainfall prediction. *J. Hydroinformatics*. **12** (4), 458–473 10.2166/hydro.2010.032 (2010).

[51] Reddy, P. C. S., Sucharitha, Y. & Narayana, G. S. Development of rainfall forecasting model using machine learning with singular spectrum analysis. *IIUM Eng. J.*, 23 (1) 172–186 https://doi.org/10.31436/IIUMEJ.V23I1.1822 (2022).

[52] X. Zhang. et al. A combined model based on secondary decomposition and the optimized support vector machine algorithm for regional rainfall forecasting *J. Water Climate Change*, 16 (1) 474-492 10.2166/wcc.2025.512 (2025).

[53] Pal Shuvo, S., Kumar Adhikary, S., Chandra Mondol, S., Sabbir Hossain, M. & Masud Rana, M. *Enhanced Prediction Of Rainfall Using A Hybrid Machine Learning Approach-A Case Study In Khulna, Bangladesh 4 Publications 0 Citations See Profile Enhanced Prediction Of Rainfall Using A Hybrid Machine Learning Approach-A Case Study In Khulna, Bangladesh.* https://www.researchgate.net/publication/379268054(2024).

[54] Mondol, S. C. et al. *A Deep Learning Approach Using Long Short-Term Memory Networks for Enhanced Prediction of Rainfall in the Northeastern Region of Bangladesh.* https://www.researchgate.net/publication/379119120 (2024).

[55] Kouadri, S., Pande, C. B., Panneerselvam, B., Moharir, K. N. & Elbeltagi, A. Prediction of irrigation groundwater quality parameters using ANN, LSTM, and MLR models. *Environ. Sci. and Pollution Res.* **29**(14), 21067–21091. 10.1007/s11356-021-17084-3 (2022). 10.1007/s11356-021-17084-3 34748181

[56] Shad, M., Sharma, Y. D. & Singh, A. Forecasting of monthly relative humidity in Delhi, India, using SARIMA and ANN models. *Model. Earth Syst. Environ.* **8** (4), 4843–4851. 10.1007/s40808-022-01385-8 (2022). 35434264 10.1007/s40808-022-01385-8 PMC8998166

[57] Geetha, A. et al. Prediction of hourly solar radiation in Tamil Nadu using ANN model with different learning algorithms. *Energy Rep.* **8**, 664–671. 10.1016/j.egyr.2021.11.190 (2022).

[58] Nur, A. S., Mohd, N. H., Radzi, & Ibrahim, A. O. Artificial Neural Network Weight Optimization: A Review. *TELKOMNIKA Indonesian J. Electr. Eng.*10.11591/telkomnika.v12i9.6264 (2014).

[59] Ho, N. X., Le, T. T., Dinh, T. H. & Nguyen, V. H. Prediction of buckling damage of steel equal angle structural members using hybrid machine learning techniques. *Sci. Rep.*10.1038/s41598-025-87869-w (2025). 39922853 10.1038/s41598-025-87869-w PMC11807133

[60] Radhi, S. M., Al-Majidi, S. D., Abbod, M. F. & Al-Raweshidy, H. S. Machine Learning Approaches for Short-Term Photovoltaic Power Forecasting. *Energies (Basel)*10.3390/en17174301 (2024).

[61] Halima, D. et al. Solar radiation Estimation based on a new combined approach of artificial neural networks (ANN) and genetic algorithms (GA) in South Algeria. *Computers Mater. Continua*. **79** (3), 4725–4740. 10.32604/cmc.2024.051002 (2024).

[62] *International Conference on Innovation and Intelligence for Informatics, Computing, and Technologies (3ICT)*. IEEE, (2018). IEEE, (2018). (2018).

[63] Bhavya, R., Sivaraj, K. & Elango, L. Ant Colony Based Artificial Neural Network for Predicting Spatial and Temporal Variation in Groundwater Quality. *Water (Switzerland)*10.3390/w15122222 (2023).

[64] Baioletti, M., Di Bari, G., Milani, A. & Poggioni, V. Differential evolution for neural networks optimization. *Mathematics*10.3390/math8010069 (2020).

[65] Lee, A., Geem, Z. W. & Suh, K. D. Determination of optimal initial weights of an artificial neural network by using the harmony search algorithm: application to breakwater armor stones. *Appl. Sci. (Switzerland)*. 10.3390/app6060164 (2016).

[66] Kuo, C. L., Kuruoglu, E. E. & Chan, W. K. V. Neural network structure optimization by simulated annealing. *Entropy*10.3390/e24030348 (2022). 35327859 10.3390/e24030348 PMC8947290

[67] Kose, U. An ant-lion optimizer-trained artificial neural network system for chaotic electroencephalogram (EEG) prediction. *Appl. Sci. (Switzerland)*. 10.3390/app8091613 (2018).

[68] Wee, W. J. et al. Application of augmented bat algorithm with artificial neural network in forecasting river inflow in Malaysia. *Appl. Water Sci.*10.1007/s13201-022-01831-z (2023).

[69] Chen, Z. et al. Egret Swarm Optimization Algorithm: An Evolutionary Computation Approach for Model Free Optimization. *Biomimetics*10.3390/biomimetics7040144 (2022). 36278701 10.3390/biomimetics7040144 PMC9590057

[70] A. A. Heidari et al. Harris hawks optimization: Algorithm and applications. *Future Gener. Comp. Syst.*. **97** 849–872. 10.1016/j.future.2019.02.028 (2024).

[71] Amiri, M. H., Mehrabi Hashjin, N., Montazeri, M., Mirjalili, S. & Khodadadi, N. Hippopotamus optimization algorithm: a novel nature-inspired optimization algorithm. *Sci. Rep.*10.1038/s41598-024-54910-3 (2024). 38424229 10.1038/s41598-024-54910-3 PMC10904400

[72] Ondrus, M., Olds, E. & Cribben, I. Factorized binary search: change point detection in the network structure of multivariate high-dimensional time series. *Imaging Neurosci.*10.1162/imag_a_00520 (2025). 10.1162/imag_a_00520 PMC12319840 40800973

[73] Shen, C., He, Y. & Qin, J. Robust Multi-Dimensional Time Series Forecasting. *Entropy*10.3390/e26010092 (2024). 38275500 10.3390/e26010092 PMC11154447

[74] Rathnayake, N., Rathnayake, U., Chathuranika, I., Dang, T. L. & Hoshino, Y. Cascaded-ANFIS to simulate nonlinear rainfall–runoff relationship. *Appl. Soft Comput.*10.1016/J.ASOC.2023.110722 (2023).

[75] Sun, W., Zhang, Y. & Chen, F. Research on heart and lung sound separation method based on DAE–NMF–VMD. *EURASIP J. Adv. Signal. Process.*10.1186/s13634-024-01152-0 (2024).

[76] Zhou, T. et al. Stable EEG Spatiospectral Patterns Estimated in Individuals by Group Information Guided NMF. *Brain Topogr*10.1007/s11269-020-02638-w (2025). 40042673 10.1007/s10548-025-01110-5

[77] Dastorani, M. T., Moghadamnia, A., Piri, J. & Rico-Ramirez, M. Application of ANN and ANFIS models for reconstructing missing flow data. *Environ. Monit. Assess.* **166** (1–4), 421–434. 10.1007/s10661-009-1012-8 (2010). 19543999 10.1007/s10661-009-1012-8

[78] Esmaeili, M., Osanloo, M., Rashidinejad, F., Aghajani Bazzazi, A. & Taji, M. Multiple regression, ANN and ANFIS models for prediction of backbreak in the open pit blasting. *Eng. Comput.* **30** (4), 549–558. 10.1007/s00366-012-0298-2 (2014).

[79] Tümer, A. E. & EDEBALİ, S. An artificial neural network model for wastewater treatment plant of Konya. *Int. J. Intell. Syst. Appl. Eng.* **3** (4), 131. 10.18201/ijisae.65358 (2015).

[80] Banik Assistant Professor, S. et al. Forecasting Bangladeshi monsoon rainfall using neural network and genetic algorithm approaches. http://www.academicglobalpublications.com/itmr/ (2009).

[81] https://earthengine.google.com/.

[82] Dananjali, T., Wijesinghe, S. & Ekanayake, J. Forecasting weekly rainfall using data mining technologies. In *2020 From Innovation to Impact, FITI 2020* (Institute of Electrical and Electronics Engineers Inc., 2020). 10.1109/FITI52050.2020.9424877.

[83] Hong, W. C. Rainfall forecasting by technological machine learning models. *Appl. Math. Comput.* **200** (1), 41–57. 10.1016/j.amc.2007.10.046 (2008).

[84] Ghosh, S., Gourisaria, M. K., Sahoo, B. & Das, H. A pragmatic ensemble learning approach for rainfall prediction. *Discover Internet Things*10.1007/s43926-023-00044-3 (2023).

[85] Baljon, M. et al. Rainfall Prediction Rate in Saudi Arabia Using Improved Machine Learning Techniques. *Water (Switzerland)*10.3390/w15040826 (2023).

[86] O. A. Wani et al. Predicting rainfall using machine learning, deep learning, and time series models across an altitudinal gradient in the North-Western Himalayas. *Sci. Rep.*10.1038/s41598-024-77687-x (2024). 39537701 10.1038/s41598-024-77687-x PMC11561348

[87] de Castro, Y. & Mencarelli, L. Forecasting nonnegative time series via sliding mask method (SMM) and latent clustered forecast (LCF), http://arxiv.org/abs/2102.05314 (2021).

[88] NMF_deri-2.

[89] Thorndike, R. L. WHO Belongs In The Family?D~.CEM ~ ER, 1953. (2019).

[90] SEVİNÇ, E. The Effect of Hidden Neurons in Single-Hidden Layer Feedforward Neural Networks. *Bilişim Teknolojileri Dergisi* **12**(4), 277–286. 10.17671/gazibtd.465886 (2019).

