# Supplementary material for: Precipitation modeling in Northeastern Bangladesh–India transboundary flood regions using bi-metaheuristic-optimized NMF-neural network
Source: Sci Rep. 2025 Oct 31;15:38166. doi: 10.1038/s41598-025-22135-7 (PMC12578858; doi:10.1038/s41598-025-22135-7)
Supplement: Supplementary file 1 — Supplementary Material 1 [file 41598_2025_22135_MOESM1_ESM.docx]

**Appendix: Metaheuristic and Probabilistic Optimization**

**Appendix A:**

**Harris Hawks Optimization algorithm (HHO)**

The Harris Hawks Optimization algorithm is a nature-inspired optimization technique proposed by Seyedali Mirjalili and his colleagues in 2019 [98]. It takes its inspiration from the cooperative hunting behavior of Harris hawks, which employ a combination of strategies to capture their prey [99]. This algorithm is renowned for its simplicity, adaptability, and efficiency, making it a popular choice for solving complex optimization problems across various fields [100]. It consists of initialization, exploration, transition, and development phases toward the best solution [101].

**Initialization Phase:** This is the phase when the positions of the hawks are initialized randomly in the solution space. The fitness value of every hawk is calculated, and the position of the best solution is checked.

**Exploration Phase:** In the Harris Hawks Optimization, the exploration phase is achieved by simulating the random stay strategy of Harris hawks during their hunting process. This process introduces a random selection factor q_r_ in the range of [0,1]. According to q_r_ values, either an exploration strategy is chosen in each iteration.

**When** $\boldsymbol{q}_{\boldsymbol{r}}\boldsymbol{\geq0.5}$**:** It is an indication that the Harris hawk moves into a random location to explore, mimicking a wider search across the solution space. This is the exploration behavior: the hawk moves to the new unexplored area, thus showing diversity in the process of searching.

**If** $\boldsymbol{q}_{\boldsymbol{r}}\boldsymbol{<0.5}$**:** The location of the hawk is decided by the location of the other family members. This will model the cooperative behavior among the hawk group, that the hawk utilizes the knowledge or the position of the other individuals in the squad to exploit promising areas.

This process introduces stochasticity in the search-such that the algorithm can balance exploration-searching new areas-and exploitation-focused on promising areas (Eq. 12).

$X(t+1)$=$\left\{ \begin{aligned} X_{rand}\left( t \right)-r_{d1} \left| X_{rand}\left( t \right)-2r_{d2}X\left( t \right) \right| q_{r}\geq0.5 \\ \left( X_{p}\left( t \right)-X_{m}\left( t \right) \right)-r_{d3}(lb+r_{d4}\left( ub-lb \right) q_{r}<0.5 \end{aligned} \right.$ ………………………………(12)

At every iteration t, where r_d1_, r_d2_, r_d3_ and r_d4_ are random numbers within the range [0, 1], the updated value influences the position update of the hawk. Then parameters, X(t), X(t+1), X_rand_(t), X_p_(t), and X_m_(t) should be updated with the iteration number t of the formula [102]. These represent the position vector of the selected hawk at the current position, the position vector of the hawk at the next iteration, a randomly selected hawk position vector, the position vector of the prey, and the average position vector of the Harris hawk population, respectively [103]. The variables ub and lb, represent upper and lower bounds. Thus, the average position vector X_m_(t) can be calculated by summing up the positions of all the hawks at the i-th iteration and averaging. This is given by Eq. 13 where N_sum_ is the total number of hawks:

$X_{m}\left( t \right)$=$\frac{1}{N_{sum}}\sum_{i=1}^{N_{sum}} X_{i}(t)$ …………………. (13)

**Transition Phase:** The run-of-sight during the transition stage depends on the variation of the prey's escape energy. The initial energy of the prey is represented as E_init_, and its value is updated during the iteration process in the range [-1, 1]. The escape energy, Etime, is defined as given in Eq. 14, where the absolute value is used to determine whether the algorithm is in either the exploration or development phase. For this, a critical value of 1 is used. If $\left| E_{time} \right|\geq1$ , the Harris hawk enters into an exploration phase. On the other hand, if, $\left| E_{time} \right|<1$ the Harris hawk enters the development phase.

$E_{time}$=2$E_{init}$(1-$\frac{t}{T}$) ……………………………………….. (14)

**Exploration phase:** It can enter the development phase only when the Harris hawk has spied its target. In this phase, a new random number g is created to give the probability that the rabbit is being preyed before the attacking of the hawk. In between the values [0, 1], threshold value is put as 0.5, $E_{time}$ and g consider the both cases and decide which attack strategy can be finalized during the development phase.

**Besiege in the Exploitation Phase:** If g$\geq0.5$ and$E_{time}\geq0.5$ , then it is assumed that the prey attempts to flee while making a sudden attack leads to being encircled. The formula in this case will be (Eq. 15- Eq. 16):

**X(t+1) =** $\boldsymbol{\Delta X}\left( \boldsymbol{t} \right)\boldsymbol{-}\boldsymbol{E}_{\boldsymbol{time}}\left| \boldsymbol{B}_{\boldsymbol{r}}\boldsymbol{X}_{\boldsymbol{p}}\left( \boldsymbol{t} \right)\boldsymbol{-X(t)} \right|$ **………………(15)**

$\Delta X(t)$=$X_{p}\left( t \right)-X(t)$ ……………….(16)

$B_{r}$=2(1- $r_{d5})$ …………………(17)

Here, $\Delta X(t)$ is the difference in distance between the prey and the hawk during the t-th iteration, d_d5_ is within the range of [0, 1], and B_r_ is the random jump strength of prey during escaping. The value of B_r_ is within the range [0, 2].

**Hard Besiege in the Exploitation Phase:** In condition when g$\geq0.5$ and $\left| E_{time} \right|<0.5$ which means that prey become surrounded increasingly, is exhausted because of the large amount of consumed energy, and is eventually captured. The following is a simulation formula (Eq. 18):

X(t+1) = $X_{p}\left( t \right)-E_{time}\left| \Delta X(t) \right|$ …………………(18)

**Soft Besiege with Progressive Rapid Dives:** In other words, if g$<0.5$ and $\left| E_{time} \right|\geq0.5$ , the Harris hawk dives. Then, this action is combined with the Levy flight (LF) function to simulate the actions of the hawk. Thus, the corresponding simulation formula becomes (Eq. 19 -Eq. 22):

Y= $X_{p}(t)-E_{time}\left| BX_{p}(t)-X(t) \right|$ ……………..(19)

Z=Y+ S×LF (dim) …………………(20)

LF(x)=0.01 × $\frac{u\times\sigma}{\left| v^{\frac{1}{\beta}} \right|}$ ……………(21)

$\sigma$=${( \frac{\Gamma(1+\beta)\times\sin\frac{\pi\beta}{2}}{\Gamma(\frac{1+\beta}{2})\times\beta\times2^{\frac{\beta-1}{2}}})}^{\frac{1}{\beta}}$ ………………(22)

Here, dim represents the dimension of the problem, while S is a random vector and the size is 1$\times dim$. LF is the Levy flight function, u and v are random numbers in the range [0, 1], and $\beta$ is fixed at 1.5 for the fight step. The updated soft besieges strategy function, after introducing the Levy flight (LF), is given by Eq. 23:

X(t+1) = $\left\{ \begin{aligned} Y if F(X)<F(X(t) \\ Z if F\left( Z \right) <F(X\left( t \right)) \end{aligned} \right.$ ………………..(23)

**Hard Besiege with Progressive Rapid Dives:** When g$<0.5$ and $\left| E_{time} \right|<0.5$ ,in the condition of hard besiege, the hawk decreases his average position interval to catch the prey that cannot escape. The simulation formula is as follows (Eq. 24):

X(t+1) = $\left\{ \begin{aligned} Y if F(X)<F(X(t) \\ Z if F\left( Z \right) <F(X\left( t \right)) \end{aligned} \right.$ …………………(24)

Under the new rules, Y and Z need to be optimized and adjusted. The simulation formula for this process is as follows (Eq. 25-Eq. 26):

Y= $X_{p}\left( t \right)-E_{time}\left| BX_{p}\left( t \right)-X_{m} (t) \right|$ …………….(25)

Z=Y+S $\times$ LF (dm) …………………………(26)

**Appendix B:**

**Hippopotamus Optimization Algorithm (HO)**

The Hippopotamus Optimization Algorithm (HO) is a step-by-step process inspired by the natural behavior of hippopotamuses. It begins with initialization, in which a herd of hippopotamuses (solutions) is randomly initialized in the search space and crucial parameters such as population size and iteration bounds are initialized [75], [104]. It is followed by aquatic movement which provides exploration by mimicking the mobility of hippos in water bodies to seek more promising locations. This is followed by the defense mechanism in which the positions of the hippos are altered based on high-fitness individuals to enable exploitation and improve solutions. To ensure diversity is maintained and premature convergence is prevented, the evasion strategy is implemented, introducing controlled randomness to mimic the evading behavior of hippos. The algorithm is completed with its termination condition, stopping when the iteration count equals its maximum or an optimum is achieved. By going through these steps quickly, HO finds a good balance between exploration and exploitation and proves to be an efficient method of solving complex optimization problems.

**Population Initialization:** The initial solution of HO is generated randomly and uses the following formula to generate a vector for the decision variable (Eq. 27):

*x_ij_ = lb_j_ + r* $\times$ *(ub_j_ - lb_j_)* (27)

Where, *x_ij_* indicates the position of *i* the candidate solution, *r* is random (0, 1), *lb_j_* and *ub_j_* represent the bounds of the *j* decision variable.

**Location Update (Exploration phase):** In the exploration phase, adult male hippopotamuses are expelled by the dominant male, and subsequently, they engage in competition with other males to gain a more favorable position. The updated position of a male hippopotamus can be calculated using the following equation (Eq. 28):

$x_{ij}^{M}=x_{ij}+y_{1}(D-I_{1}x_{ij})$ (28)

Here, x_ij_^M^ denotes the male hippopotamus's new position, y_1_ is a random number between 0 and 1, and D represents the dominant hippopotamus's position. The integers *I*_1_ and *I*_2_ are within the range of [1, 2]

*h*= $\left\{ \begin{aligned} I_{2}\times r_{1}+(\sim Q_{1}) \\ 2\times r_{2}-1 \\ \overset{\to}{r_{3}} \\ I_{1}\times r_{4}+({\sim Q}_{2}) \\ r_{5} \end{aligned} \right.$ (29)

T= exp (-$\frac{1}{T}$) (30)

$x_{ij}^{FB}=\left\{ \begin{aligned} x_{ij}+h_{1}\left( D-I_{2}MG_{1} \right),T>0.6 \\ E,else \end{aligned} \right.$ (31)

$E=\left\{ \begin{aligned} x_{ij}+h_{2}\left( MG_{i}-D \right),r_{6}>0.5 \\ {lb}_{j}+r_{7}\left( {ub}_{j}-{lb}_{j} \right),else \end{aligned} \right.$ (32)

Additional equations define the movement of immature or female hippopotamuses within the population, where random numbers control the updates. For instance, the values r_1_ through r_7​_ represent random values that influence the position updates. The objective function value, F_i_, helps determine if a particular position should be accepted or rejected.

$x_{i}=\left\{ \begin{aligned} x_{i}^{M},F_{i}^{M}<F_{i} \\ x_{i},else \end{aligned} \right.$ (33)

$x_{i}=\left\{ \begin{aligned} x_{i}^{FB},F_{i}^{FB}<F_{i} \\ x_{i},else \end{aligned} \right.$ (34)

Equations (33) and (34) are used to describe the final positions of male hippopotamuses and other vulnerable individuals. If the updated objective function value Fi is improved, the position is adjusted accordingly.

**Hippo Defense against Predators (Exploration Phase):** Vulnerable hippopotamuses in the group can wander away from the herd and be an easy prey for major predators. The position of these individuals is updated according to the following equation:

$P_{j}={lb}_{j}+r_{8}({ub}_{j}-{lb}_{j})$ (35)

$D=\left| P_{j}-x_{ij} \right|$ (36)

Where, P_j​_ represents the position of the hippopotamus, and r_8_ is a random value between 0 and 1. Equation (35) describes the distance from a predator to the hippopotamus. If the distance is small, the hippopotamus will adopt defensive behavior based on F_Pj​_ to protect itself. If F_Pj_ is smaller than the objective function value F_i_​, this indicates a higher risk of predation, prompting the hippopotamus to approach the predator and force it to retreat. If F_Pj ​_ is larger, Equation (37) suggests the predator is farther away, and the hippopotamus will stay within its territory to deter the predator without approaching it.

$x_{ij}^{HR}=\left\{ \begin{aligned} R_{L}\bigoplus P_{j}+\left( \frac{f}{\delta-d\cos\left( 2\pi y \right)} \right)⦁\left( \frac{1}{D} \right),F_{Pj}<F_{i} \\ R_{L}\bigoplus P_{j}+\left( \frac{f}{\delta-d\cos\left( 2\pi y \right)} \right)⦁\left( \frac{1}{2\times D+r_{9}} \right),F_{Pj}<F_{i} \end{aligned} \right.$ (37)

*x_ij_^HR^* is the posture of a hippopotamus when facing predators, *R_L_* represents changes in the position of the predator when attacking hippopotamuses. The calculation formula for the Levy distribution is Equation (37). *ω* and *υ* are random [0, 1], *σ_ω_* can be obtained by calculating Equation (39).

$L(v)=0.05\times\frac{\omega\times\sigma_{\omega}}{|{v|}^{\frac{1}{v}}}$ (39)

In Equation (37), *f* is random [2, 4], *d* is random [1, 1.5], *D* is random [2, 3], *δ* is random [−1, 1]. *r*_9_ is an m-dimensional random vector. From Equation (40), if *F_i_^HR^* is larger than *F_i_*, represents the position of the hippopotamus will be replaced; otherwise, the hippopotamus will return to the population.

$x_{i}=\left\{ \begin{aligned} x_{i}^{HR},F_{i}^{HR}<F_{I} \\ x_{i},F_{i}^{HR}\geq F_{i} \end{aligned} \right.$ (40)

**Hippo Escape from Predators (Development Phase):** When hippopotamuses encounter predators, they may employ defensive strategies to move away and escape danger. To model this escape behavior, a position is selected at random near the current position of the hippopotamus. This is done many times over, with the aim of obtaining a safer position. If the newly found position results in a lower objective function value (F_i_), it indicates that the hippopotamus has successfully identified a safer location and will update its position accordingly.

${lb}_{j}^{local}=\frac{{lb}_{j}}{t},{ub}_{j}^{local}=\frac{{ub}_{j}}{t},t=1,2,\ldots..,T$ (41)

$x_{ij}^{HE}=x_{ij}+r_{10}\left( {lb}_{j}^{local}+s_{1}\left( {ub}_{j}^{local}-{lb}_{j}^{local} \right) \right)$ (42)

In equation (42),$x_{ij}^{HE}$ is to search for the location of the hippopotamus to find the nearest safe location, s_1_ selected from Equation (43). The scenario under consideration has stronger local search capabilities.

$s_{1}=\left\{ \begin{aligned} 2\times r_{11}-1 \\ r_{12} \\ r_{13} \end{aligned} \right.$ (43)

In Equation (44), *r*_11_ Represents a random vector [0, 1], *r*_10_ and *r*_13_ are random [0, 1], *r*_12_ is a random variable that follows a normal distribution.

$x_{i}=\left\{ \begin{aligned} x_{i}^{HE},F_{i}^{HE}<F_{i} \\ x_{i},F_{i}^{HE}\geq F_{i} \end{aligned} \right.$ (44)

**Appendix C:**

**Egret Swarm Optimization Algorithm (ESOA):**

Egrets are categorized as Great Egret, Middle Egret, Little Egret, and Yellow-billed Egret, all being white-plumed birds that inhabit wetlands and coastal areas. Great Egrets pursue larger prey actively, whereas Snowy Egrets utilize a sit-and-wait method, conserving energy but capturing prey more frequently. Egret Swarm Optimization Algorithm (ESOA) is the one that incorporates these methods of foraging, which is both energy efficient and proactive exploration. It contains three strategies: discriminant condition, sit-and-wait, and aggressive search. ESOA mimics natural hunting behavior by assigning roles to three egret species to create optimal exploration and optimization. It better evades the pitfalls of conventional optimization methods compared to other techniques. [**Fig. 15**](#F15) shows the roles and the search behaviors of the Egret Squad [105].

**Sit-and-Wait Strategy:**

**Observation Equation:** The position of the i-th egret squad is denoted as x_i_ $\in R^{n}$, with n being the dimensionality of the problem. The function A (*) is Snowy Egret's estimation method to assess the possible availability of prey in its current position. Variable $\hat{y}$ estimates the availability of prey at this location (Eq. 45– 46).

**Fig. 15:** Roles and the Search Behaviors of the Egret Squad

$\hat{y}_{i}$= A (x_i_), …………………………………….(45)

$\hat{y}_{i}$= w_i_.x_i_ ………………………………………..(46)

Where, w_i_ refers to the weight. The error e_i_ could be described as Eq. 47.

e_i_ = $\frac{\left\| \hat{y}_{i}-y_{i} \right\|^{2}}{2}$ …………………………………(47)

$\hat{g}_{i}\epsilon R^{n}=$ practical gradient of $w_{i} (Eq. 48),$can be retrieved by taking the partial derivative of $w_{i}$ for the error Equation 47, and its direction is $\hat{d}_{i}$ (Eq. 49)

$\hat{g}_{i}= \frac{\partial\hat{e}_{i}}{\partial\hat{w}_{i}}$ = $\frac{\partial\frac{\left\| \hat{y}_{i}-y_{i} \right\|^{2}}{2}}{\partial w_{i}}$ = ($\hat{y}_{i}-y_{i}). x_{i}$ …………………..( 48)

$\hat{d}_{i}$= $\frac{\hat{g}_{i}}{\left| \hat{g}_{i} \right|}$ ………………………………………………..(49)

$\hat{d}_{h,i}$= $\frac{x_{\mathrm{ibest}}-x_{i}}{\left| x_{\mathrm{ibest}}-x_{i} \right|}$. $\frac{f_{\mathrm{ibest}}-f_{i}}{\left| x_{\mathrm{ibest}}-x_{i} \right|}$ + $d_{\mathrm{ibest}}$ …………………….(50)

$\hat{d}_{g,i}$= $\frac{x_{\mathrm{gbest}}-x_{i}}{\left| x_{\mathrm{gbest}}-x_{i} \right|}$. $\frac{f_{\mathrm{gbest}}-f_{i}}{\left| x_{\mathrm{gbest}}-x_{i} \right|}$ + $d_{\mathrm{gbest}}$ ……………………(51)

$\hat{d}_{h,i}$ is the directional correction of the best location of the squad (Eq. 50) while $\hat{d}_{g,i}$ is the directional correction of the best location of all squad (Eq. 51). The gradient $g_{i}$ can be represent as Eq. 52.

$g_{i}$= (1-$r_{h}-r_{g}). \hat{d_{i}}$ + $r_{h}.d_{h,i}+r_{g}.d_{g,i}$ ………………….(52)

An adaptive weight update method is applied here (Eq. 53) [76], $\beta_{1}is 0.9$ and $\beta_{2}$ is 0.99:

$m_{i}$=$\beta_{1}.m_{i}+\left( 1-\beta_{1} \right).g_{i}$

$m_{i}$=$\beta_{1}.m_{i}+\left( 1-\beta_{1} \right).g_{i}$ (53)

$v_{i}$=$\beta_{1}.m_{i}+\left( 1-\beta_{1} \right).{g^{2}}_{i}$

$w_{i}$= $w_{i}-\frac{m_{i}}{\sqrt{v_{i}}}$

Based on the judgment that Egret A makes of the current situation, the next sampling location $x_{a,i}$ is described as Eq. 54. $y_{a,i}$ (Eq. 55) presents the fitness of $x_{a,i}$.

$x_{a,i}$= $x_{i}$ + $\mathrm{step}_{a}.\exp(\frac{-t}{0.1.t_{\max}})). hop. g_{i}$ …………………. (54)

$y_{a,i}$=f($x_{a,i})$………………………………………………….(55)

**Aggressive Strategy:**

Egret B is the model performing a random search for prey for widespread exploitation and to avoid falling into a local optimum within the environment. Such a mechanism for random search would provide a possibility for the algorithm of global exploration in solution space for newer, more diversified solutions. This random wandering lets Egret B allow the algorithm not to lose its capability of exploration to avoid quick, probably suboptimal convergence at solutions. That makes it a stochastic movement pattern that enhances the overall adaptability and effectiveness of the strategy that Egret Squad follows Eq. 56 and Eq. 57.

$x_{b,i}$= $x_{i}+{step}_{b}. tan\left( r_{b,i} \right). \frac{hop}{(1+t)}$ ………………………………..(56)

$y_{b,i}$=f ($x_{b,i})$ ……………………………………………………….(57)

The random search behavior of Egret B is mathematically modeled where r_b,i_ is a random number within the range ($\frac{\pi}{2}, \frac{\pi}{2}$), x_b,i_ is the expected position of Egret B, and y_b,i_ indicates the fitness value. On the contrary, Egret C uses an aggressive strategy for pursuing prey, adopting an encircling mechanism for position updating. This ensures that Egret C focuses its search efforts on refining and narrowing the area around a better solution to enhance the accuracy of the solution. In this way, Egret B's random exploration and Egret C's targeted encircling behavior balance and make the search process effective.

$D_{h}$= $x_{ibest}$-$x_{i}$ ………………………………………….(58)

$D_{g}$= $x_{gbest}$-$x_{i}$ …………………………………………(59)

$x_{c,i}$= (1-$r_{i}-r_{g}).x_{i}+ r_{h}.D_{h}+r_{g}.D_{g}$ …………………(60)

$y_{c,i}$=f ($x_{c,i})$ ……………………………………………..(61)

In the searching mechanisms of the Egret Squad, D_h_ ( Eq. 58) is the gap matrix between the current position and the best position in the specific Egret squad, and D_g_ (Eq. 59) compares the current position against the best among all the positions of Egret squads. These matrices help with the calculation of the distance of the squad or individual egrets from the optimal solution. For Egret C, x_c,i_ (Eq. 60) is its expected next location based on its aggressive encircling strategy. The step size factor step_b_$\in(0,1]$ within the range of 0 to 1 determines how far Egret B moves in each step of its random search, which controls the exploration behavior. Further, r_h_ and r_g_ are random numbers in the range [0, 0.5), which add stochasticity to the search process. These random factors prevent the algorithm from falling into deterministic behavior, ensuring that the search for optimal solutions is dynamic and effective. These variables altogether contribute to the balanced behavior of the Egret Squad in combining exploration and exploitation efficiently.

**Discriminant Condition:**

After each member of the Egret squad has determined its private plan according to its strategy, that is, random search for Egret B, encircling for Egret C, or guiding for Egret A, the squad as a whole chooses the best option among those plans. The squad takes the joint action by applying the selected strategy and moves the solution toward better solution. The solution matrix represents the solution of the i-th Egret squad, recording the position or state of the squad at a certain point in the search process (Eq. 62). In this way, the above matrix is used to memorize and update the collective decisions and movements of the squad; hence, the group makes coordinated steps toward the optimal solution. The Egret Squad maximizes the possibility of the best solution by integrating efforts from all squad members through individual and group action.

$x_{s,i}$=$\left[ \begin{matrix} x_{a,i} & x_{b,i} & x_{c,i} \end{matrix} \right]$ ………………(62)

$y_{s,i}$=$\left[ \begin{matrix} y_{a,i} & y_{b,i} & y_{c,i} \end{matrix} \right]$ ……………….(63)

$c_{i}$=argmin ($y_{s,i})$ …………………..(64)

$x_{i}$=$\left\{ \begin{aligned} x_{s,i/ci} \\ x_{i} \end{aligned} \right.$ …………………………..(65)

If the minimal value of y_s,i_ (the fitness value of the i-th Egret squad) is better than the current fitness y_i_, the Egret squad accepts the new plan and moves to the updated solution (Eq. 63). In other words, the new solution will be accepted provided that its fitness value is superior to the previous one; otherwise, the same position remains unchanged. That is to say, it is a 30% chance that the random number between 0 and 1 could be less than 0.3, based on which, the squad can still accept an inferior plan. This makes the procedure a little randomized and adds an exploration aspect to the choice-making procedure, allowing it to sometimes take risks for the chance at better solutions later on. This strategy allows the algorithm to avoid being stuck in a local optimum and further favors exploration in the solution space.

**Appendix D:**

**Genetic Algorithm (GA)**

Genetic Algorithm (GA) represents a class of optimization methods with roots in the principles of natural selection and evolutionary biology as originally proposed in the 1970s by John Holland [106]. The algorithms imitate biological processes: reproduction, mutation, crossover, and selection to be able to find or approximate the exact optimal solution for complex problems.

**Population Initialization:** Individuals are represented in a real-coded manner. Every individual is a real-valued string composed of four parts: the weights between the input layer and the hidden layer, the thresholds of the hidden layer, the weights between the hidden and the output layer, and the thresholds of the output layer [107]. These parts put together carry all the weights and thresholds of the neural network. Given the known structure of the network, on any given mapping specified by nodes, weights, and thresholds, a neural network can be built [108].

**Fitness Function:** According to the initial values of the BP neural network based on individuals, train the network by the provided training data and predict the system output. Then, calculate every individual's fitness F, which is defined as the absolute error and variance E between the estimated output and the expected output. The fitness can be determined as follows Eq. 66:

F= k$(\sum_{i=1}^{n} abs(y_{i}-o_{i}))$ ……………..(66)

where n is the number of the network's output nodes; yi is the expected output of the i-th node. $o_{i}$ is the predicted output of the i-th node; k is the coefficient for normalization.

**Selection operation:** Selection operation in GA follows the strategy of fitness proportion selection. The probability of selection of an individual is directly proportional to the fitness of the individual. Selection probability p_i_ for every individual i is computed as Eq. 67- Eq. 68:

$f_{i}=\frac{k}{F_{i}}$ ………………(67)

$p_{i}=\frac{f_{i}}{\sum_{j=1}^{N} f_{j}}$ ………………(68)

The fitness value of individual i, denoted as $F_{i}$, reflects its quality. Since it is desired that the smaller the value is, this fitness value will be inverted before selection. Here, k is the coefficient with the same value defined in Eq. 70, and N represents the total number of individuals in the population.

**Crossover operation:** Because the individuals are represented in real encoding, a real-number crossover method should be used for the crossover operation. For the k-th chromosome a_k_ and the l-th chromosome a_l_, at position j the crossover is realized by Eq. 71-Eq. 72.

$a_{kj}=a_{kj}\left( 1-b \right)+a_{lj}b$ ………….(69)

$a_{lj}=a_{ij}\left( 1-b \right)+a_{kj}b$ …………….(70)

where: b is a random number within (0, 1).

**Mutation Operation:** The j-th gene of the i-th individual, denoted as a_ij_, is chosen for mutation. The mutation operation is performed according to the formula (Eq. 71- Eq. 72):

$a_{ij}=a_{ij}+\left( a_{ij}-a_{max} \right)\times f\left( g \right) r>0.5$ ………………(71)

$a_{ij}=a_{ij}+\left( a_{min}-a_{ij} \right)\times f\left( g \right) r\leq0.5$ ……………….(72)

Where, $a_{max}$ is the upper bound for the gene $a_{ij}$. $a_{min}$is the lower bound for the gene$a_{ij}$. f(g)=r_2_${(1-\frac{g}{G_{max}})}^{2}$ ,where r2 is a random number between 0 and 1. g is the current iteration count. G_max_ is the maximum number of iterations allowed for evolution. r is another random number within the range (0, 1), which is automatically generated when a_ij_ is selected to determine whether the mutation operation is executed.

**Appendix E:**

**Particle Swarm Optimization**

Particle Swarm Optimization is a newly developed optimization technique in the realm of evolutionary computation, which is inspired by the social aspect of bird flocking or schooling fish [109], [110]. This was developed from observing how individuals in a group cooperate and share information to accomplish a common objective, such as finding food or migrating efficiently [111]. The PSO algorithm is based on two basic equations [112], [113]. The first, called the velocity equation, is given by Eq. 73, which describes how the velocity of each particle should be updated [114]. A particle updates its velocity based on its personal best position, the swarm's global best position, and the current position of the particle [115], [116]. The coefficients c1 and c2 are the acceleration factors that express the individual and social influences of the particle, respectively.

These are usually some coefficients which can also be interpreted as trust parameters defining the degree of confidence of a particle: c1 - reliance of a particle on itself and c2 - reliance on neighbors, together with random factors r1 and r2 introduce the stochastic ingredient into simulated cognitive and social dynamics.

$v_{i}^{t+1}$= $v_{i}^{t}$ + $c_{1}r_{1}\left( {pbest}_{i}^{t}-p_{i}^{t} \right)+ c_{2}r_{2}({gbest}^{t}-p_{i}^{t})$ …………(73)

Where, $v_{i}^{t}$= Inertia, $c_{1}r_{1}\left( \mathrm{pbest}_{i}^{t}-p_{i}^{t} \right)=Personal influence$, $c_{2}r_{2}\left( \mathrm{gbest}^{t}-p_{i}^{t} \right)$= Social influence

The second, Eq. 74, is the position equation where each particle refreshes its position by using the newly calculated velocity:

$p_{i}^{t+1}$= $p_{i}^{t}+ v_{i}^{t+1}$ …………..(74)

The parameters of position and velocity are interdependent, i.e., the velocity depends on the position and vice-versa.

**Appendix F:**

**Bayesian optimization (BO):**

Bayesian optimization is a sequential, stochastic optimization method used to minimize expensive black-box functions where function evaluation is computationally expensive [117]. Bayesian optimization techniques make use of constructing a surrogate model, usually a Gaussian Process (GP) utilized to approximate the objective function based on previously observed samples [118]. A pair of random function samples is initially executed and a GP is fitted on observations that estimate the function's mean and uncertainty. An acquisition function, such as Expected Improvement (EI) or Upper Confidence Bound (UCB), is then used to determine the next sampling point by balancing exploration (sampling in the less certain region) and exploitation (sampling at where the function is most expected to be optimal). This is done iteratively, incorporating each new evaluation into the GP model and constricting the search for the global optimum. This efficient sampling technique allows Bayesian optimization to find the best solutions within a limited number of function calls, making it highly applicable for hyperparameter tuning in machine learning and other optimization problems.

Transfer function and number of hidden neurons optimization in an Artificial Neural Network (ANN) is a systematic search for the best combination that enhances model performance. The procedure begins by defining an objective function, say the minimization of Mean Squared Error (MSE) or the maximization of R², to compare different configurations. The ANN is trained for each configuration on a specified training set and tested on a different validation set to measure its performance without risking overfitting. The search algorithm repeatedly modifies the parameters, evaluating the performance of the model and selecting the configuration yielding the best outcomes. After the optimal transfer function and number of neurons are determined, the model is tested against a separate test set to ensure that it can be generalized. Optimization here ensures that the ANN is precise and yet computationally efficient.
